# Transcriptome analysis of bolting in *A. tequilana* reveals roles for florigen, MADS, fructans and gibberellins

**DOI:** 10.1186/s12864-019-5808-9

**Published:** 2019-06-10

**Authors:** Emmanuel Avila de Dios, Luis Delaye, June Simpson

**Affiliations:** Department of Genetic Engineering, Cinvestav Unidad Irapuato, Km. 9.6 Libramiento Norte Carretera Irapuato-León, Apdo. Postal 629, 36821 Irapuato, Guanajuato Mexico

**Keywords:** RNAseq, *A. tequilana*, Bolting, Flowering locus T, MADS, Fructan, Gibberellins

## Abstract

**Background:**

Reliable indicators for the onset of flowering are not available for most perennial monocarpic species, representing a drawback for crops such as bamboo, agave and banana. The ability to predict and control the transition to the reproductive stage in *A. tequilana* would represent an advantage for field management of agaves for tequila production and for the development of a laboratory model for agave species.

**Results:**

Consistent morphological features could not be determined for the vegetative to reproductive transition in *A. tequilana*. However, changes in carbohydrate metabolism where sucrose decreased and fructans of higher degree of polymerization increased in leaves before and after the vegetative to reproductive transition were observed. At the molecular level, transcriptome analysis from leaf and shoot apical meristem tissue of *A. tequilana* plants from different developmental stages identified OASES as the most effective assembly program and revealed evidence for incomplete transcript processing in the highly redundant assembly obtained. Gene ontology analysis uncovered enrichment for terms associated with carbohydrate and hormone metabolism and detailed analysis of expression patterns for individual genes revealed roles for specific Flowering locus T (florigen), MADS box proteins, gibberellins and fructans in the transition to flowering.

**Conclusions:**

Based on the data obtained, a preliminary model was developed to describe the regulatory mechanisms underlying the initiation of flowering in *A. tequilana*. Identification of specific promoter and repressor Flowering Locus T and MADS box genes facilitates functional analysis and the development of strategies to modulate the vegetative to reproductive transition in *A. tequilana*.

**Electronic supplementary material:**

The online version of this article (10.1186/s12864-019-5808-9) contains supplementary material, which is available to authorized users.

## Background

To date most studies related to regulation of the vegetative to reproductive transition have focused on monocarpic annual species such as *A. thaliana* or polycarpic perennial species such as poplar and data is lacking for monocarpic perennials such as *Agave*. The ability to undergo prolonged vegetative growth periods implies that these species ignore the seasonal changes in temperature and photoperiod to which the annual and polycarpic species respond, until reaching a point where endogenous and environmental stimuli converge to initiate the transition to the reproductive phase [1]. This process is of particular interest in *Agave* species which are exploited as crop plants for the production of tequila and mezcal in Mexico, for fiber production in Africa, China and Brazil [2–5] and are being developed as bioenergy sources in Australia [6, 7]. Initiation of flowering signals the optimum stage for harvesting of agaves, and the inflorescence is manually removed in the early stages of development to avoid rapid depletion of fructan reserves. Time of flowering within a single plantation can differ by a few days or several years and *Agave* producers must opt to either leave immature plants to be harvested in successive years in order to optimize sugar content or harvest all plants within a field simultaneously where many will not have reached maturity. On the other hand, long life cycles and the perennial, monocarpic mode of growth of *Agave* species has hampered breeding and genetic analysis. In *Agave* species, onset of bolting can be identified by a phenomenon called “sinking” or “flattening” (Additional file 1). However, umbel meristems are present at this stage indicating that the conversion of the SAM to an inflorescence meristem has already occurred [8]. The possibility to manipulate flowering time to optimize harvesting or alternatively to produce early flowering genotypes would greatly facilitate commercial production and basic research.

In *A. thaliana*, an annually flowering species, [9] transition to the reproductive phase is regulated by 5 different pathways: ageing, gibberellin, autonomous, day-length and vernalization, controlled by environmental or endogenous factors. These pathways converge on a phosphatidylethanolamine binding protein (PEBP) denominated Flowering locus T (FT) synthesized in phloem companion cells of leaf tissue and transported through the phloem to the shoot apical meristem where it interacts with other proteins to promote the transition to flowering by regulating the activity of MADS box proteins [9].

Synthesis of FT is strictly controlled and depends on the correct combination of inputs from the 5 pathways. Analysis of FT in other plant species has shown that PEBP/FT proteins are encoded by gene families which vary in the number of members and based on specific amino acid configurations may either promote or repress flowering [9–11].

The hormone gibberellin (GA) also stimulates the transition to flowering in *A. thaliana* and may stimulate FT expression or act independently to promote flowering under long days [12, 13]. Movement of GA to the SAM has also been demonstrated in *Lolium* and it has been suggested that both FT and GA constitute the mobile florigen signal [9]. However, studies in perennial species [14, 15] have shown that application of GA inhibits flower induction and promotes vegetative growth [16–18].

In addition to the 5 pathways mentioned above, increasing evidence indicates a role for carbohydrates in the regulation of the vegetative to reproductive transition not just as energy reserves for inflorescence growth but also as signaling molecules. Although starch, fructans, trehalose and sucrose have all been implicated in the signaling mechanism, the precise role of these metabolites and their relation to the 5 defined flowering pathways is not yet well defined [19].

In order to identify the morphological and biochemical factors involved in the initiation of bolting in *A. tequilana*, in this work morphological traits for plants of different ages and growth stages and carbohydrate profiles were compared in plants sampled in fields managed by different tequila producers. To understand the regulation of this process at the molecular level the Illumina NextSeq™ 500 platform was employed to generate RNAseq data and compare the transcriptomes of leaves and shoot apical meristems (SAM) from vegetative and reproductive growth stages. Results indicated that morphological traits alone are inconsistent descriptors to define the vegetative to reproductive transition whereas carbohydrate analysis showed profiles consistent with the age of the plants. RNAseq data revealed specific metabolic processes and genes that are preferentially expressed in leaf or SAM tissue during the transition and led to detailed analysis of the Flowering locus T and MADS box gene families and genes associated with fructan and gibberellin metabolism, representing a first step towards the prediction and control of the vegetative to reproductive transition in *A. tequilana*.

## Methods

### Plant material and RNA isolation

Samples obtained from shoot apical meristems (SAM) and leaves (mid-section of the third layer of fully opened leaves below the SAM, (Additional file 1) of 4, 6 and 7 year old *A. tequilana* plants were collected from commercial fields of Tequila Sauza, in the region of Los altos de Jalisco, Mexico. Plants of 4 years were in the vegetative stage of growth, whereas plants of 6 years were at the initial stage of bolting/sinking (Additional file 1) reported by Delgado-Sandoval [8], on the other hand plants of 7 years had an inflorescence of 1 m (1MI). All tissues were collected in triplicate, frozen in liquid nitrogen, and stored at − 80 °C until processed. Tissues were ground to a fine powder using a mortar and pestle and Total RNA was isolated using the TRIzol reagent (Invitrogen) and the PureLink RNA Mini Kit Purification System (Invitrogen) according to the manufacturer’s protocol.

### Measurements in commercial fields

Measurements were obtained from *A. tequilana* plants of 2, 4, (vegetative stage, V) and 6 (sinking stage, S) years of age from 60 plants in commercial fields of Tequila Sauza and 33 plants of 6 years of age in a commercial field of Tequila Real de Penjamo. The number of leaves, number of suckers, height and diameter were recorded for each plant.

### Detection of simple sugars and fructans

Soluble sugars were extracted by grinding green leaf samples in liquid nitrogen followed by freezing and lyophilization of the samples until a constant weight was achieved. 200 mg of lyophilized tissue were then resuspended in 30 ml of water at 75 °C for 30 min. The water was removed and stored at 4 °C and the sample resuspended in 20 ml of water at 75 °C for 15 min. Water was again removed and the samples were combined and frozen before concentration by lyophilization. Quantification of sucrose, glucose and fructose in the samples was carried out using the K-SUFRG kit (Megazyme) and the manufacturers instructions. Presence and estimation of DP of fructans was carried out using the kit K-FRUC kit (Megazyme) and by TLC as described in [20].

### Generation of RNA-seq data

Both library preparation and cDNA sequencing were performed at the Laboratory of Genomic Services UGA-LANGEBIO, Cinvestav, Mexico. Indexed TruSeq Libraries for three biological replicates from each tissue and developmental stage were sequenced on the Illumina NextSeq™ 500 platform in a Single End 136 base run.

### Raw data processing *and* de novo assembly

Raw Illumina reads were processed with Trimmomatic 0.36 [21] to remove adapter sequences from the reads with low quality and shorter than 50 nucleotides. The remaining reads were considered of high quality and were used to build the transcriptome. De Novo assembly was carried out with three different assemblers, Trinity v20140413 [22], Trinity 2.2.0 and Oases [23] 0.2.08 together with Velvet [24, 25]. Trinity v20140413 assemblies were carried out with default parameters, assemblies generated by Trinity 2.2.0 used kmer 25 and kmer 31, whereas the Oases assembly was performed initially through four assemblies, using kmer 44 from 77 with steps of 12 nt and the results were combined to create a single assembly. For comparisons, Oases contigs shorter than 200 nt were deleted and Cd-hit [26, 27] was used to generate clusters of 99.9% identity and reduce the redundancy of the assembly. To compare the different de novo assemblies, contig characteristics such as N50, median, number of contigs, number of contigs with BLASTx hit and BUSCO results were calculated.

### Transcriptome annotation

Blast alignments were carried out with BLASTx and the contigs were compared against TAIR10 and Plant Refseq databases. The alignments were considered as significant only for E-values ≤10^− 5^ and a Score ≥ 69 with TAIR alignments taking priority over Refseq due to the better annotation on the *Arabidopsis thaliana* database. Gene Ontology annotation was retrieved from the Panther database.

### Prediction of non-coding RNAs

To predict non-coding RNAs, contigs without a significant Blastx were processed with the Coding-Potential Assessment Tool and Coding Protein Calculator [28, 29] where only contigs with a CPAT score ≤ 0.3 and CPC score ≤ − 1 were considered as non-coding RNAs.

### Identification of differentially expressed clusters

Statistical analysis was carried out in R [30] ambient and EdgeR was used to determine which genes were overexpressed in contrasting conditions, only genes with a FDR ≤ 0.01 were considered as significant. Due to the highly redundant nature of the transcriptome and evidence for retention of introns, cd-hit was used to generate clusters with at least 50% of aligned bases with 95% of identity to the longest sequence of clusters. On average, 75% of high quality reads could be mapped against the 155,485 clusters generated. These clusters were then used to generate in silico expression data.

### Gene ontology enrichment analysis

Enrichment of gene ontology (GO) terms was carried out in the agriGO web server [31], the differentially expressed clusters with GO annotation for each comparison were used as query and the complete set of contigs with GO annotation was used as background. Only terms of complete GO with at least 5 mapping entries and a FDR ≤ 0.05 were reported. For a results summary and visualization REVIGO [32] was used with allowed similarity set to small.

### Identification and characterization of the amino acid sequences of candidate genes

Identification of sequences, reconstruction of cDNAs, determination of amino acid sequences and construction of dendrograms was carried out essentially as described in Avila de Dios et al. 2015 [20].

## Results

### Morphological characteristics of *A. tequilana* plants of different ages

**Fig. 1:**
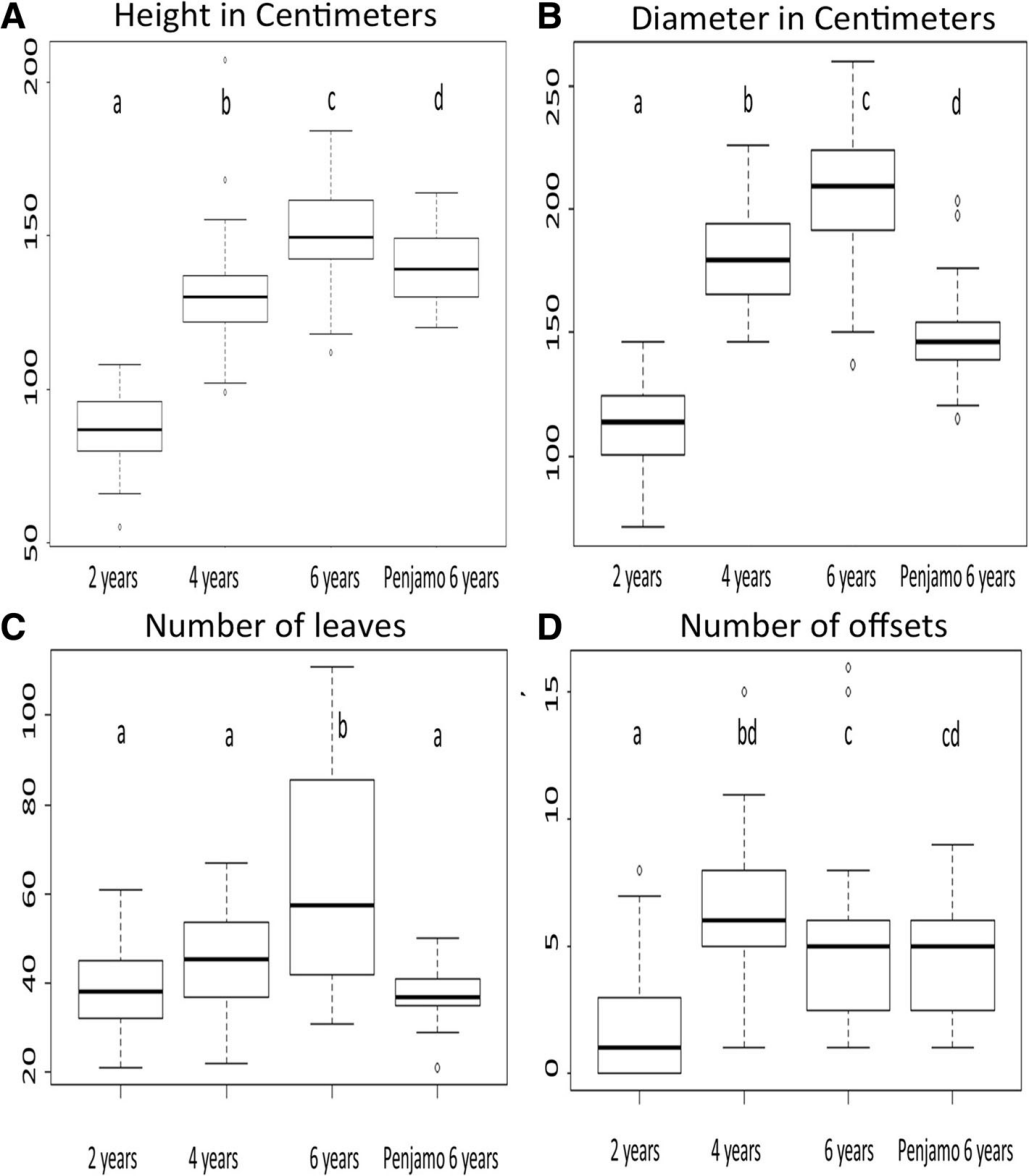
Morphological parameters of *A. tequilana* plants sampled in commercial fields of two Tequila companies at different growth stages. Letters indicate a significant difference between in each group

To determine whether the age and onset of bolting of *A. tequilana* plants in commercial fields could be estimated based on morphological parameters, height, diameter, number of leaves and number of suckers was recorded for plants of 2, 4 and 6 years of age from plantations of two different tequila companies: “Real de Penjamo” (RdP) and “Tequila Sauza” (TS). A significant increase in plant height was observed according to age (Fig. 1a). However a significant difference in height was also observed between 6 year old plants sampled in commercial fields managed by different tequila producers, where RdP plants were considerably smaller than TS plants. A similar pattern was found when the diameter was analyzed, increasing according to plant age for TS plants. However the diameter of 6-year-old RdP plants was even smaller than 4-year-old TS plants (Fig. 1b). An overall increase in the number of leaves in relation to age was observed for TS plants although the number of leaves for each plant varied widely, especially for 6-year-old plants (Fig. 1c). A striking difference was observed between 6 year old plants from TS in comparison to 6-year-old plants from RdP where in the latter fields, 6-year-old plants had a similar number or even fewer leaves as those of 2-year-old plants from TS (Fig. 1c). The number of suckers per plant was lowest for 2 year old plants from TS but similar numbers were recorded for 4 and 6-year-old TS plants and 6-year-old RdP plants (Fig. 1d).

### Sugar content in leaf tissue of *A. tequilana* plants of different ages

**Fig. 2:**
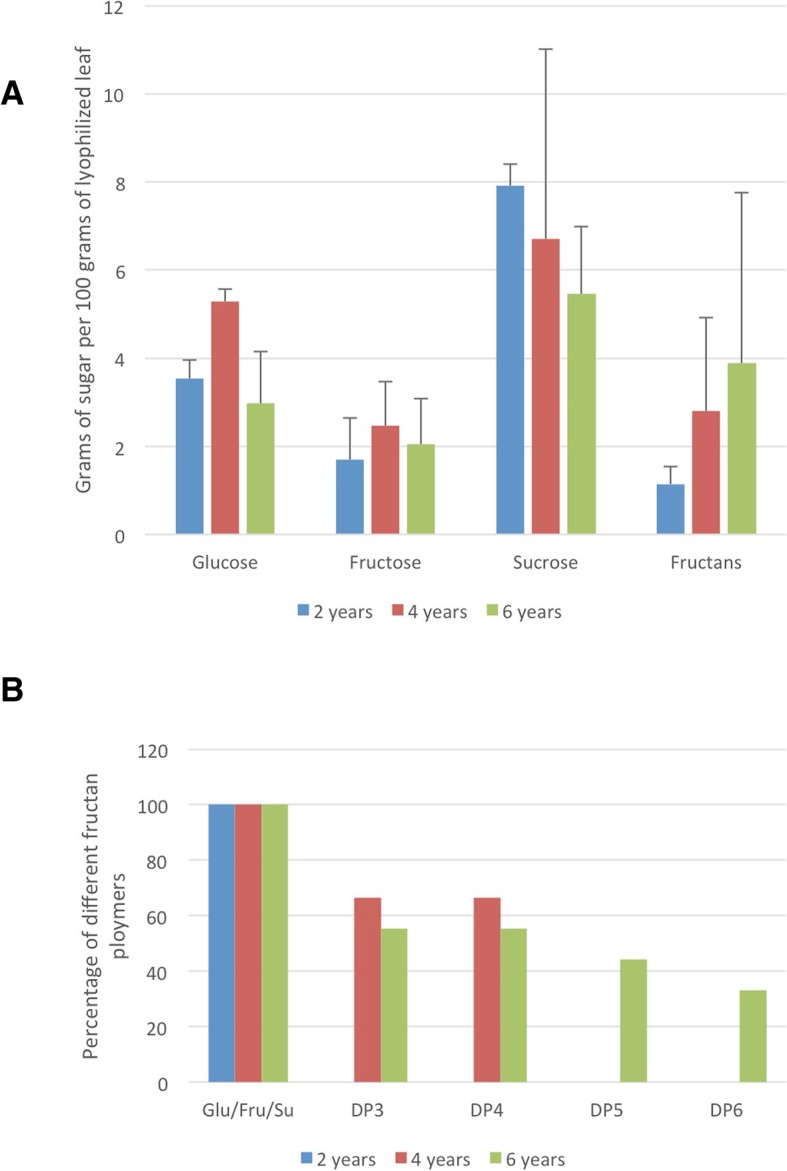
Accumulation of carbohydrates in leaves of *A. tequilana* plants of different ages. **a**. Levels of different sugars present per 100 g of lyophilized tissue. Colors of bars indicate the age of the sampled plants. **b**. Percentage of different fructan polymers in leaves of plants of different ages. Colors of bars indicate the age of the sampled plants

Although the level and degree of polymerization (DP) of fructans in *A. tequilana* stem tissue in relation to plant age has been reported previously [33–35], no data is available for leaf tissue. To determine whether the levels of simple sugars or fructans in leaf tissue were correlated to plant age and the process of bolting, the presence of glucose, fructose, sucrose and fructans were determined in the mid-section of leaves of *A. tequilana* plants of 2, 4 and 6 years of age. Figure 2 A shows that no significant difference was observed for glucose, fructose or sucrose in leaves from plants of different ages. However the overall level of sucrose decreased with plant age whereas fructan levels increased. Fructans with degrees of polymerization (DP) 3 and 4 were detected in leaves of 4-year-old plants and up to DP6 in 6-year-old plants (Fig. 2b).

### Comparison of RNA-seq de novo assembly methods

More than 610 × 10^6^ reads with a length of 136 nt from 18 indexed libraries were generated from leaves and meristems of *A. tequilana* plants at the vegetative (V), initiation of bolting (S) and 1 m inflorescence stages (I1M) (Additional file 1). On average 34 × 10^6^ sequences for meristem tissues and 33 × 10^6^ sequences for leaf tissues were obtained. After removing low quality sequences, 491,948,259 quality reads (80.5% of total) were used to reconstruct the transcriptome.

For de novo assembly the results obtained using Trinity 2.2.0, Trinity v20140413 and Oases 0.2.08 were compared to identify the best assembly procedure. Among the three Trinity assemblies, the lowest number of contigs, longest average length and N50 length was obtained by Trinity v20140413 (Table 1). Due to similar N50, average length and median between Trinity 2.2.0 with kmer 31 and Trinity v20140413 assemblies, but a greater capacity to reconstruct longer protein coding transcripts Trinity 2.2.0 was chosen as “the best” Trinity assembly.

**Table 1:** Comparison of Contig characteristics for the assemblies generated

Assembly	Trinity v20140413	Trinity 2.2.0 kmer 25	Trinity 2.2.0 kmer 31	Oases 0.2.08 Kmer 31–77
Contigs	371,885	593,947	562,115	490,956
MB assembled	305	415	445	705
Average length	822 nt	698 nt	793 nt	1437 nt
Median	442 nt	404 nt	444 nt	1042 nt
N50	1446 nt	1089 nt	1326 nt	2184 nt
%GC	40.77	41.28	41.34	41.28
Blastx TAIR hits> 70%	10,921	11,370	11,708	12,566

*MB* Megabases, *nt* nucleotides

Oases assemblies were combined automatically using the script Oases_pipeline.py and fewer contigs were obtained in comparison to the best Trinity assembly (Table 1). In total 858 proteins missed in the best Trinity assembly were obtained with the Oases assembly and this method was selected for all further analyses. To compare the Oases transcriptome obtained with a previous *A. tequilana* transcriptome [36] generated from vegetative stage tissues, BUSCO was used to identify single copy orthologs. Both transcriptomes are similar (Additional file 2) with only 73 BUSCOs identified in the previous transcriptome remaining unidentified in the present work and 53 identified in this work unidentified previously. Interestingly most of the BUSCOs identified in the current analysis were reported as duplicated rather than single copies.

### The highly redundant assembly contains many incompletely processed mRNAs

**Fig. 3:**
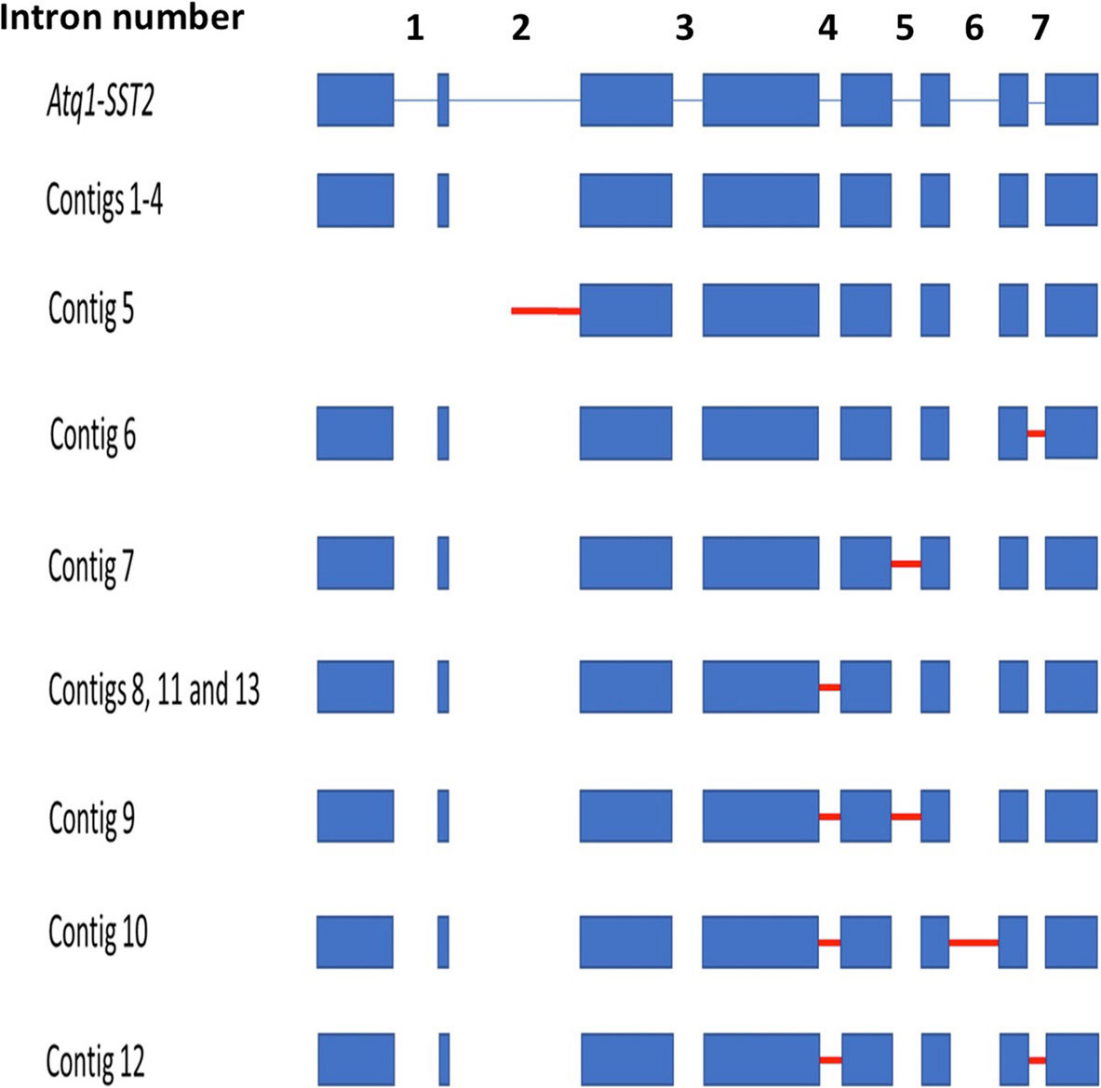
Alignment scheme for Atq1-SST2 with the 13 different contigs. Blue boxes indicate exons, blue lines represent introns and red lines represent introns retained in each contig. Numbers designated to each intron are indicated above the figure

The lack of a reference genome for *Agave* species makes it difficult to predict whether the transcriptome redundancy observed reflects the complexity of the transcription process in *A. tequilana* in the stages analyzed, whether it is an effect of the assembly process or a combination of both possibilities. With the aim of addressing this problem, genomic sequences available for enzymes of Plant Glycoside Hydrolase Family 32 [37] were chosen to identify the presence of differentially processed mRNAs by determining the numbers of contigs associated with individual genes. For the *Atq1-SST2* gene, 12 contigs containing the whole ORF and 1 partial contig lacking the 5’region including the first 2 exons (Fig. 3, Additional file 3) were identified. Four contigs representing the expected completely processed mRNA for Atq1-SST2 and differing only slightly in the 3′ UTR region were identified. Contig 5 skips the first two exons and retains part of second intron and the other 7 contigs show different combinations of retention of at least one of the introns numbered 4–7 (Fig. 3). No contigs retaining introns 1 and 3 were identified. All contigs could potentially be translated to produce proteins of varying length and percentage identity in relation to the reported sequence and may represent different levels of enzyme activity.

### Transcriptome annotation

Contig alignments using Blastx against the TAIR10 protein database uncovered 240,330 significant contigs representing only 48.9% of the total number. A second Blastx against the Refseq Plant database, using the contigs with no significant hits to TAIR, revealed a further 36,866 contigs with significant hits (7.5% of the total).

**Fig. 4:**
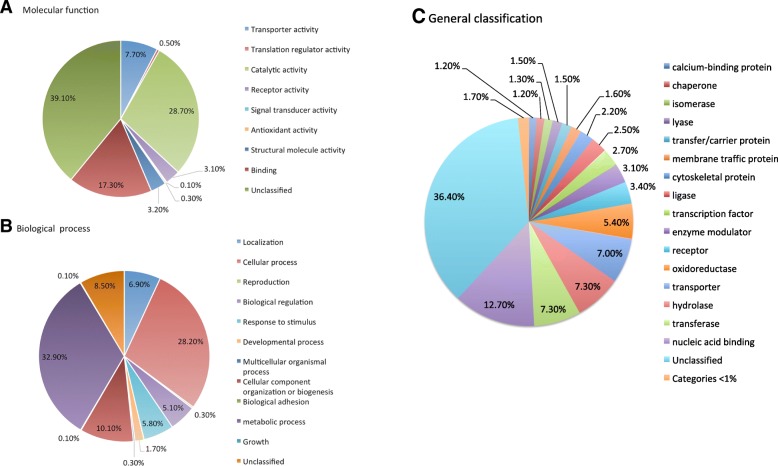
Functional annotation of transcriptome sequences based on Gene Ontology terms. **a**. Molecular function, **b**. Biological process and **c**. General classification, for the *A. tequilana* transcriptome

For functional annotation, TAIR accession numbers obtained by Blastx were mapped against the Panther database and represent a general snapshot of the information currently available for the transcriptome of *A. tequilana*. When “molecular function” was retrieved (Fig. 4a), the top three categories were Catalytic activity, Binding and Transporter activity. When “biological process” was retrieved the top three categories were Metabolic process, Cellular process and localization (Fig. 4b). Similarly, a general classification of proteins was obtained where the principal categories were: Nucleic acid binding, transferases, hydrolases and transporter proteins (Fig. 4c).

### Prediction of non-coding RNAs

CPAT and CPC analyses with stringent criteria predicted 161,218 contigs containing non-coding RNAs representing 32.8% of the total number of contigs assembled (Additional file 4). In total 52,542 contigs could not be classified and were catalogued as unknown contigs. Contigs classified as non-coding have the lowest GC content whereas those encoding mRNAs have the highest and unclassified RNAs have an intermediate value (Additional file 4).

### In silico analysis of differential expression

A heat diagram of overall expression patterns of each individual replica for each tissue and developmental stage shows a clear separation of leaf and meristem samples and tissue and developmental stage indicating that the data obtained are consistent and robust, (Additional file 5).

### Enrichment of transcripts overexpressed in leaves and SAM during bolting

To identify key changes in gene expression relating to the vegetative to reproductive transition, in silico expression patterns were analyzed in detail by determining clusters overexpressed in leaves and meristems between the V and S and S and 1MI stages of development and vice versa. To summarize and visualize the results of the GO enrichment REVIGO was used (Additional files 6, 7, 8 and 9). In V stage leaves 248 clusters were overexpressed in comparison S stage leaves and 28 enrichment terms including: cell wall organization or biogenesis and phenylpropanoid metabolic process were identified. A similar number of clusters (221) were found to be overexpressed in the leaves of S stage plants however a higher number (66) of GO enrichment terms were determined. Of these, 23 were related to: response to endogenous and exogenous stimulus, catabolic process, and aging. In SAM tissue the number of overexpressed clusters was around 13 fold higher, where 3218 clusters were overexpressed in V stage SAM and 3350 clusters in S stage SAM. Enrichment analysis for the overexpressed V stage SAM clusters showed 260 enrichment terms and similarly to vegetative leaves, most of them are related to developmental process, homeostasis, or biosynthetic process. Interestingly, regulation of gibberellic acid mediated signaling pathway is an enrichment term for these clusters.

Among 342 enrichment terms determined for S stage meristems, 25 of them are related to responses to stimulus, whereas carbohydrate metabolic process and photosynthesis are other interesting terms. Collectively, results from leaves and meristems from S plants suggest a role for carbohydrates in the vegetative to reproductive transition in *A. tequilana*. In S leaves, 418 clusters were overexpressed and the enrichment analysis identified 109 enrichment terms whereas 1147 overexpressed clusters were identified in leaves of the 1MI stage. For S clusters, enriched terms included: regulation of reproductive process, photosynthesis and generation of precursor metabolites and energy suggesting these processes are more important in leaves at the early stages of the reproductive transition in *A. tequilana* plants. This is also reflected in the overexpression results from the S stage SAM tissue where 2505 clusters representing 386 GO enriched terms were determined, with reproductive structure development and reproduction being the most important terms. For 1MI SAM, 2846 clusters were overexpressed. Unlike the overexpressed clusters in S tissues, the enrichment terms for the 1147 clusters overexpressed in leaves and 2846 in meristems from 1MI tissues include more general terms or terms not specifically related to flowering. For instance, in leaves, cell wall organization and plant-type secondary cell wall biogenesis are important and this may reflect the fact that leaves from plants in the reproductive stage begin to senesce. Similarly, in meristems of 1MI plants, almost 40% of the enrichment terms were associated with response to stimulus and grouped in the cluster of response to temperature stimulus**.** Whereas similar numbers of overexpressed clusters were identified for the same tissue type in the other comparisons, the number of overexpressed clusters identified for 1MI leaf tissue is almost 3 fold higher than for S leaf tissue**.** A summary of the overexpression/enrichment analysis is presented in Table 2.

**Table 2:** Summary of overexpression/enrichment analyses

Developmental stages	No. overexpressed clusters	No. enriched GO terms
LEAVES		
V/S	248	28
S/V	221	66
S/1MI	418	109
1MI/S	1417	214
SAM		
V/S	3218	260
S/V	3350	342
S/1MI	2505	386
1MI/S	2846	170

Vegetative (V), initiation of bolting (S) and 1 m inflorescence stages (I1M)

### Detailed analysis of selected differentially expressed genes in leaf and SAM tissue at V, S and 1MI developmental stages

#### FT family genes

**Fig. 5:**
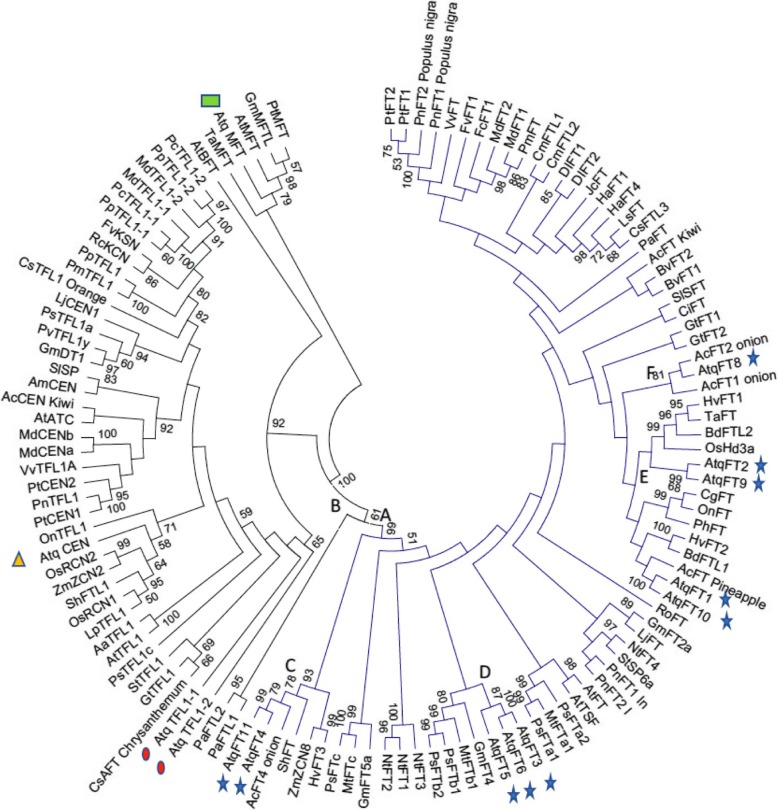
Phylogenetic relationships between the *A. tequilana* FT family and other plant species. Blue lines indicate all FT type genes. Blue stars indicate *A. tequilana* FT genes. Black lines indicate other FT family members. The green square shows *A. tequilana* “Mother of FT gene”, the yellow triangle indicates the *A. tequilana* Centroradialis gene and red ovals indicate *A. tequilana* terminal flower genes

Given the importance of the Flowering Locus T gene family for the process of bolting in other plant species, the FT family was characterized for *A. tequilana*. In total, 10 full-length cDNAs and 1 partial cDNA could be reconstructed for FT. Two cDNAs were determined for Terminal Flower (TFL) type genes, one cDNA corresponding to a putative Mother of FT gene and one to a Centroradialis type gene. Alignment of the corresponding amino acid sequences for each complete cDNA with corresponding protein sequences from other species confirmed the initial classification as shown in the dendrogram in Fig. 5. Based on this distribution, the *A. tequilana* FT genes identified have been named AtqFT1–11 (Additional file 10). From the specific amino acid sequences in highly conserved motifs, FT genes can be predicted to either promote or inhibit the transition to the reproductive phase and the completion of flowering. From this analysis (Additional file 11), 6 genes were predicted to promote flowering, 3 to repress flowering and one to have a possible weak TFL activity (Table 3). AtqFT7 was not included in the analysis since a full-length sequence could not be determined.

**Table 3:** Predicted functions of FT genes identified in *A. tequilana*

FT protein	Position of amino acid residues that define FT function					Predicted function
	89	138	144	146	Triad(I/L)YN	
FT1	FT	FT inducer	FT inducer	FT inducer	Conserved	FT
FT2	FT	FT inducer	FT inducer	FT inducer	Conserved	FT
FT3	FT	FT inducer	FT inducer	FT inducer	Conserved	FT
FT4	FT	Repressor FT	Repressor FT	Neither FT or TFL1	Non- Conserved	Repressor FT
FT5	FT	Repressor FT	FT inducer	FT inducer	Non- Conserved	Repressor FT
FT6	TFL1	FT inducer	FT inducer	FT inducer	Conserved	TFL1
FT7	Pseudogene					
FT8	FT	FT inducer	FT inducer	FT inducer	Conserved	FT
FT9	FT	FT inducer	FT inducer	FT inducer	Conserved	FT
FT10	FT	FT inducer	FT inducer	FT inducer	Conserved	FT
FT11	FT	Repressor FT	Repressor FT	Neither FT or TFL1	Non- Conserved	Repressor FT

**Fig. 6:**
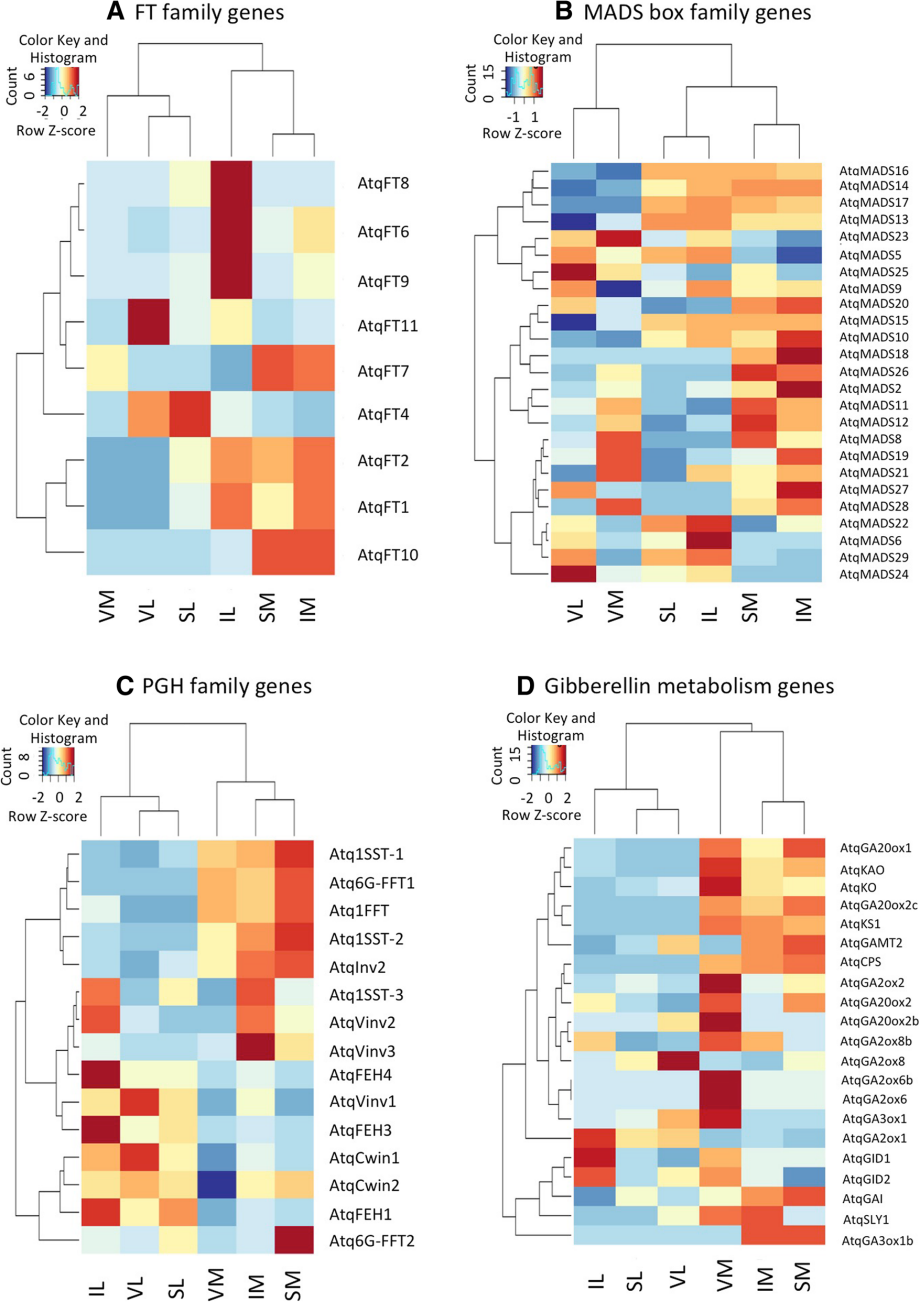
Heat diagrams of in silico gene expression patterns. **a**. FT genes, **b**. MADS box genes, **c**. PGHF32 Genes, **d**. Gibberelin metabolism genes. Red indicates higher numbers of transcripts whereas blue indicates lower numbers of transcripts

**Fig. 7:**
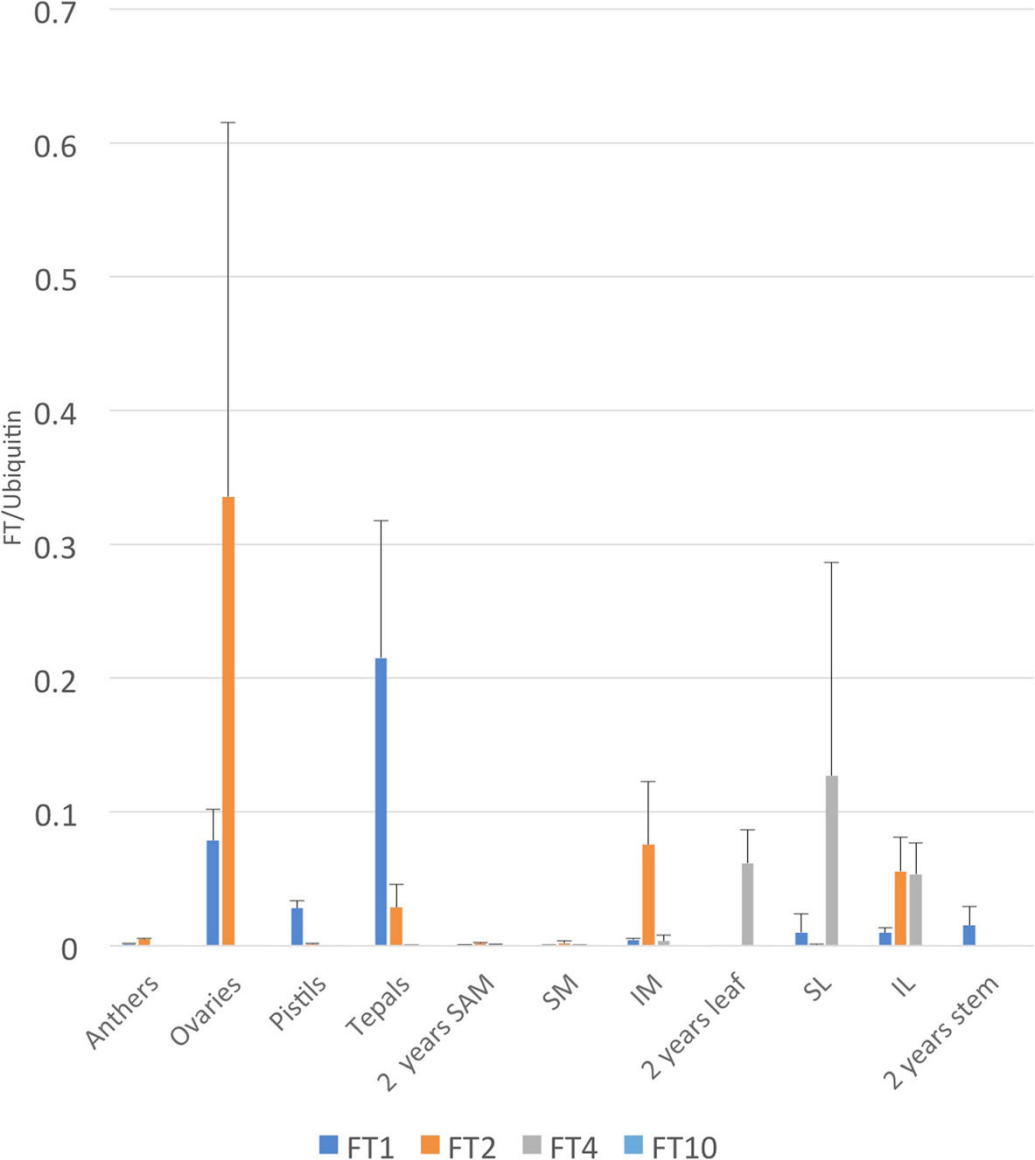
qRT-PCR analysis of FT gene expression in different tissues and at different developmental stages and in different tissues. Colors indicate different *A. tequilana* FT genes: Blue-AtqFT1, orange-AtqFT2, grey-AtqFT4 and pale blue-AtqFT10

In silico expression patterns were obtained for the FT family genes and are presented in the heat map in Fig. 6a. Interestingly differences were observed between vegetative and reproductive stages where the predicted repressor type genes AtqFT4 and AtqFT11 are strongly expressed in vegetative leaves and to some extent in leaves at the reproductive stages but not in vegetative or reproductive meristems suggesting that these genes may be responsible for the repression of flowering at the vegetative stage. In contrast, genes predicted to be flowering promoter type FTs AtqFT 1, 2,6, 9, and 10 are expressed at low levels in vegetative tissues but strongly expressed in reproductive meristems at both stages suggesting that these genes are responsible for the induction and maintenance of the reproductive phase. AtqFT10 is not expressed in leaf tissue at the reproductive stages, however transcripts for all other genes of both repressor and promoter type were found in this tissue suggesting that the modulation of FT protein may depend on a balance between the repressor and promoter type genes or that once bolting is initiated, these genes are no longer strictly regulated in leaf tissue. In order to obtain support for these hypotheses, qRT-PCR was carried out for specific tissues and developmental stages for 3 promoter-type FTs (AtqFT1, 2, and 10) and one repressor-type FT (AtqFT4). The results confirm the expression patterns observed for both the promoter and repressor type genes and also revealed strong expression of promoter type FTs in specific floral organs such as ovaries and tepals but not in anthers or pistils (Fig. 7).

### MADS box family genes

**Fig. 8:**
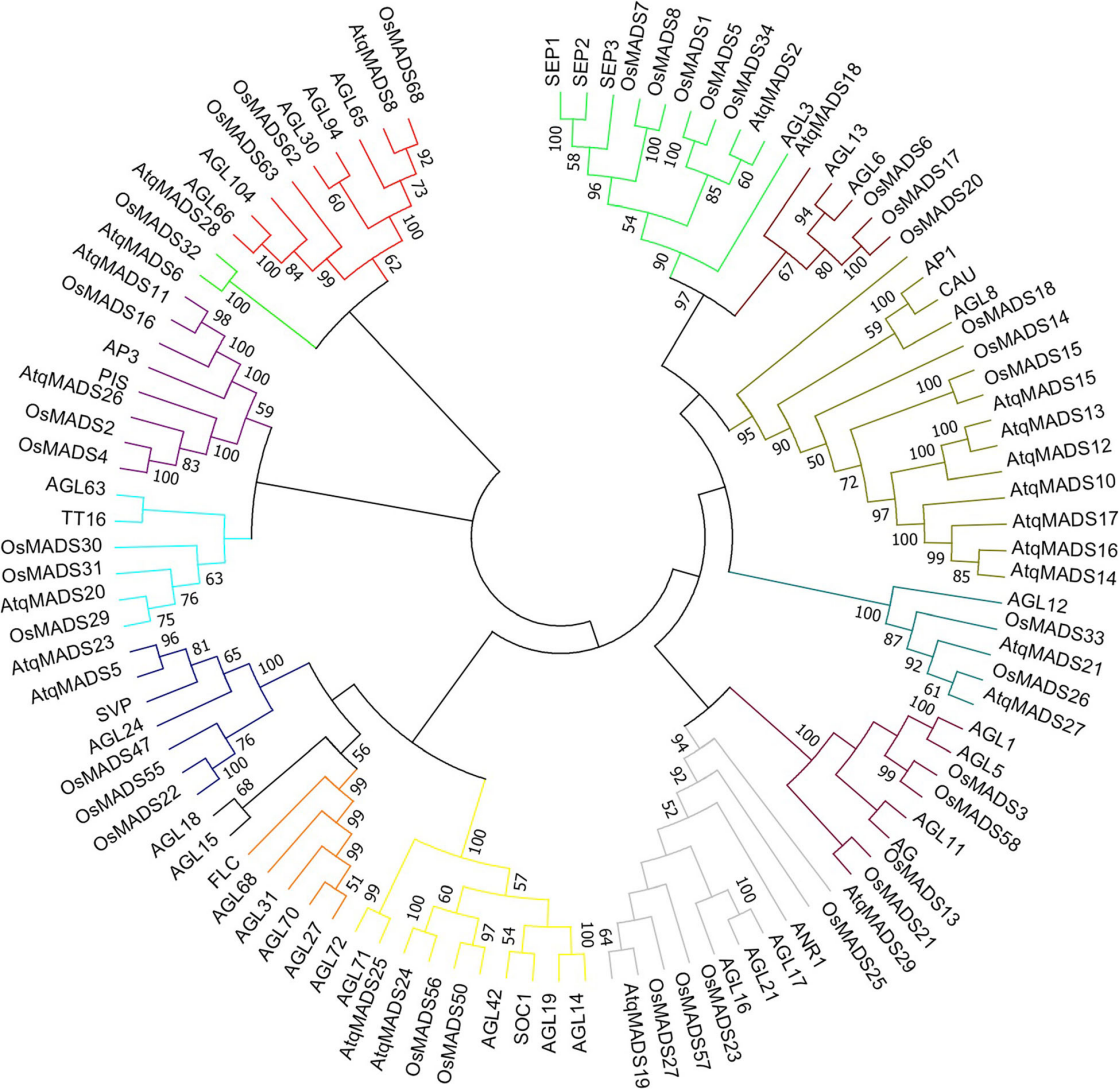
Phylogenetic relationships between the *A. tequilana* MADS class II genes and those identified in other plant species. Colors indicate distinct clades of MADS class II genes described for *A. thaliana* or other plant species

In a previous report Delgado-Sandoval et al. [8] characterized 7 MADS box genes from *A. tequilana* and showed differential expression patterns at different developmental stages and in different tissues. To confirm and extend this study, MADS genes were also analyzed in the new transcriptomes developed in this report. A total of 25 distinct MADS genes were identified (Additional file 12) including 3 genes from the previous study. Two were classified as MADS Type I: AtqMADS9 Subclade α and AtqMADS22, Subclade γ and 23 as MADS Type II (Table 4, Fig. 8). No *A. tequilana* genes were identified for the Agamous (AG), FLC or AGL15 clades but AtqMADS28 was determined to be a member of the CFO

**Table 4:** Classification of *A. tequilana* MADS based on relation to *A. thaliana* MADS

*A. tequilana* MADS	Length in aa	Type *(A. thaliana)*	Subtype *(A. thaliana)*	*A. tequilana* MADS	Length in aa	Type *(A. thaliana)*	Subtype *(A. thaliana)*
AtqMADS2^+^	263	II	SEP	AtqMADS17	249	II	AP1
AtqMADS5^+^	225	II	SVP	AtqMADS18	240	II	AGL6
AtqMADS6^+^	227	II	AP3	AtqMADS19	240	II	ANR1
AtqMADS8	350	II	MIKC*	AtqMADS20	239	II	TT16
AtqMADS9	306	I	Mα	AtqMADS21	225	II	AGL12
AtqMADS10	283	II	AP1	AtqMADS22	222	I	Mγ
AtqMADS11	220	II	AP3	AtqMADS23	213	II	SVP
AtqMADS12	238	II	AP1	AtqMADS24	170	II	SOC
AtqMADS13	241	II	AP1	AtqMADS25	211	II	SOC
AtqMADS14	259	II	AP1	AtqMADS26	184	II	AP3
AtqMADS15	229	II	AP1	AtqMADS27	201	II	AGL12
AtqMADS16	252	II	AP1	AtqMADS28	196	II	MIKC*
				AtqMADS29	194	II	ANR1

+ *A. tequilana* MADS genes reported previously, aa-amino acids

clade described previously in gramineous species. Interestingly 7 of the 23 Type II genes encoded Apetala 1 (AP1) related MADS proteins and all shared a similar in silico expression pattern (Fig. 6b) where they were more highly expressed in leaf and SAM tissue from plants in the reproductive phase in comparison to leaf and SAM tissue from vegetative stage plants. This is consistent with the role determined for AP1 as a floral meristem identity-defining gene upregulated by FT in *A. thaliana*. The two *A. tequilana* genes determined to encode SVP-like MADS proteins (AtqMADS5 And AtqMADS23), also showed consistent although slightly different expression patterns in relation to their role as repressors of FT in leaves before the transition to flowering is initiated, as determined in *A. thaliana*. AtqMADS23 showed strong expression in vegetative leaf and SAM tissue but low expression in leaves and meristems of reproductive stage plants. AtqMADS5 showed expression in leaf tissue from both vegetative and reproductive stages but not in SAM tissue at any stage. The genes identified as homologues of the *A. thaliana* SOC1 gene, AtqMADS24 and AtqMADS25 show strong expression only in vegetative leaf tissue. This is in contrast to results from *A. thaliana* that show that SOC1 is activated by FT in SAM tissue to promote the transition towards the reproductive stage. The gene denominated AtqMADS18 encodes an AGL6 type MADS protein and was specifically expressed in reproductive stage SAM tissue.

### Genes involved in fructan metabolism

The GO enrichment analysis revealed the importance of carbohydrate metabolism in the vegetative to reproductive transition and previous reports from our group have shown the importance of enzymes in Plant Glycoside Hydrolase Family 32 (PGHF32, involved in fructan and sucrose metabolism) [20] in floral tissue and starch metabolism in meristems associated with actively growing tissues [38]. Only 1 new PGHF32 gene (Atq1SST-3) was identified in this study (Additional file 10). Detailed analyses of the expression patterns of genes encoding PGHF32 enzymes (Fig. 6c) showed a clearly differential pattern of expression between leaf and meristem tissue and between the majority of genes for biosynthetic enzymes (Atq1-SST1, Atq1-SST2, Atq6GFFT1 and Atq1FFT1) or genes encoding enzymes for fructan degradation (AtqFEH1, 3 and 4). Whereas genes involved in synthesis are strongly expressed in meristem tissue in comparison to leaves and show highest expression in S stage meristems, the FEH encoding genes are strongly expressed in leaves but show low expression in meristems, suggesting that fructans are synthesized and accumulated in meristem tissue but not in leaf tissue where they are degraded. Two genes involved in fructan synthesis (Atq6G1FFT2 and At1SST-3) show distinct patterns of expression. Atq1-SST3 is mainly expressed in meristems and leaves after the vegetative to reproductive transition at the S and 1MI stages whereas Atq6G1FFT2 is strongly expressed in meristems at the S stage. Cell wall and vacuolar invertases are also members of PGHF32 and were also included in the analysis, however the genes encoding these enzymes show individual patterns of expression in different tissues and developmental stages and no clear association with bolting can be determined (Fig. 6c).

### Gibberellin metabolism

Gibberellin (GA) has been shown to induce or inhibit flowering depending on the growth habit of the plant species. The GO enrichment analysis of transcriptome data indicated a role for GA in the vegetative to reproductive transition *in A. tequilana*. A total of 10 genes for the synthesis and 7 for the degradation or inactivation of gibberellins, were identified in the transcriptome data (Tables 5 and 6, Additional file 10). The in silico expression patterns obtained for genes involved in GA metabolism or encoding GA receptor proteins uncovered interesting results suggesting that this growth regulator is carefully regulated in SAM tissue during this process. As in the case of carbohydrate metabolism, clear differences in expression patterns between leaf and SAM tissue were observed for genes involved in GA metabolism. However GA metabolism genes also show a differential pattern of expression between SAM tissue at different developmental stages (Fig. 6d). In the vegetative SAM almost all GA associated genes are strongly expressed, however in SAM tissue at the S and 1MI stages, GA catabolism genes are downregulated in comparison to GA biosynthesis genes. This suggests that GA turnover is highly regulated in vegetative SAM, whereas catabolism of GA is reduced in reproductive SAM tissue.

**Table 5:** Genes encoding proteins involved in the synthesis of gibberellins

Locus /name *A. thaliana*	Length of Protein aa	Name *A. tequilana*	Length of Protein aa
AT4G02780/CPS	802	AtqCPS	Partial, 752
AT1G79460/KS1	785	AtqKS1	764
AT5G25900/ KO	509	AtqKO	509
AT2G32440/ KAO2	489	AtqKAO	509
AT4G25420/GA20ox1	377	AtqGA20ox1a	389
AT5G51810/GA20ox2	378	AtqGA20ox2	390
		AtqGA20ox2b	381
		AtqGA20ox2c	464
AT1G15550/ GA3ox1	358	AtqGA3ox1a	364
		AtqGA3ox1b	340

*aa* amino acids

**Table 6:** Genes encoding proteins involved in the degradation/inactivation of gibberellins

Locus /name *A. thaliana*	Length of Protein aa	Name *A. tequilana*	Length of Protein aa
AT1G78440/ GA2ox1	329	AtqGA2ox1	332
AT1G30040/ GA2ox2	341	AtqGA2ox2	331
AT1G02400/ GA2ox6	329	AtqGA2ox6	353
		AtqGA2ox6b	332
AT4G21200/ GA2ox8	338	AtqGA2ox8	327
		AtqGA2ox8b	329
AT5G56300/ GAMT2	383	AtqGAMT2a	387

*aa* amino acids

## Discussion

Based on morphological traits no clear correlation with plant age or capacity for flowering was determined when commercial fields from two tequila factories were compared. This can be attributed to factors such as: use of suckers of different sizes and ages, differences in agronomical practices between producers and differences in environmental conditions between plantations. Although the Tequila Regulatory Council (CRT) recommends planting suckers of the same size and age, in practice diameters can range from 8 cm to > 15 cm, leading to heterogeneous flowering within a single plantation (https://www.youtube.com/watch?v=vRBtkU51VRI). Agronomical factors including: density of planting, levels of application of agrochemicals, removal of lower leaves and removal of suckers in addition to environmental variation will also impact on growth and time to maturity. Morphological traits alone are therefore unreliable for the delimitation of bolting in *A. tequilana*.

Differences in sugar concentration in *A. tequilana* [35] and changes in expression of genes encoding enzymes involved in carbohydrate metabolism in stem tissue have been reported in plants of different ages [37]. The novel data on leaf tissue presented here also shows a pattern of accumulation of fructans of higher degrees of polymerization (dp) and decreasing levels of sucrose in mature leaves in comparison to young leaves, indicating a dynamic carbohydrate metabolism that correlates with plant age. Data suggest that starch metabolism however does not play an important role in this process. Since *A. tequilana* plants do not respond to annual fluctuations in temperature and photoperiod during the vegetative phase, environmental factors must act in combination with factors that determine plant age in order to prevent precocious flowering following the first winter. Carbohydrates can promote bolting through the age-related down-regulation of miR156 and the concomitant up-regulation of flowering promoters [39, 40]. The accumulation of specific higher dp fructan polymers and the decrease in sucrose content observed may also lead to age-related regulation of miRNAs in *A. tequilana*.

Previous reports of transcriptome analysis in Agave species [36, 41, 42] have employed several assembly methods. Therefore, different assembly programs were compared and the Oases assembly was chosen as optimum since it allowed prediction of a greater number of annotated contigs.

The Oases assembly also showed redundancy of contigs and inspection of corresponding genomic sequences revealed retention of different intron combinations for members of PGHF32. Redundancy could be due to a low rate of RNA maturation, alternative splicing or a combination of both. Another plausible explanation is that a paleopolyploidy event in the *Agave* genus [43], led to an increased number of genes and transcript redundancy. In the example shown, only contig number 5 would produce a protein lacking the β-fructosidase domain essential for fructosyltransferase activity and would therefore be inactive. Notably, 7 of the 9 contigs showing intron retention included intron 4 either alone or in combination with other introns and may be significant in terms of enzymatic activity. Alternative splicing has previously been reported for genes encoding members of PGHF32 [44], therefore it will be interesting to determine whether the observed retention of introns has a biological effect on fructan metabolism or is related to a specific tissue, developmental stage or the age of the plant.

Overall annotation, prediction of non-coding RNAs and determination of the percentage of contigs of unknown function are comparable with other transcriptome studies and results from individual replicas indicate that the data is robust. Enrichment analyses determined that SAM tissue shows higher levels of differentially expressed genes and enriched GO terms in comparison to leaves, probably reflecting the active growth in this tissue. Numbers of overexpressed clusters were similar for comparisons between leaf tissue at V, S and 1MI stages, however the number of enriched GO terms for each comparison differed widely. In general enriched GO terms were consistent with the growth stages analyzed and notably responses to cold, heat and carbohydrates were identified supporting the hypothesis that carbohydrate metabolism in combination with environmental factors is involved in the regulation of bolting in *A. tequilana*. GO terms determined for the S stage in relation to the vegetative stage for leaf tissue suggest that levels of gene expression and the types of genes expressed are relatively similar in both these stages and that S stage leaves are already undergoing the changes relevant to the final stage of their lifecycle. Similar results were observed for the S/1MI and 1MI/S comparisons in leaf tissue where a 2-fold higher and 3-fold higher difference respectively in the number of overexpressed clusters were determined, including terms associated with cell wall biogenesis, carbohydrate metabolism, phenylpropanoid metabolism and responses to stimuli and chitin possibly reflecting that 1MI stage leaves are already undergoing the transcriptional changes related to senescence.

In contrast to leaf tissue, comparisons between SAM tissue at the different developmental stages showed between 2 and 15 fold higher numbers of overexpressed clusters in comparison to leaf tissue and numbers of enriched GO terms varied between 170 and 386. These results reflect the dynamic nature of SAM tissue consistent with the developmental changes leading to the reproductive transition. Comparisons of V and S stage meristems indicate a heightened metabolic activity in the initial stage of the transition (V/S) that decreases as the reproductive phase and growth of the inflorescence become established (S/1MI). This is reflected in the associated enriched GO terms: metabolism, ribosomes, carbohydrates, cofactor metabolism, proton transport and regulation of biological processes among others.

One enriched term in V SAM stage in relation to S stage SAM, “regulation of gibberellic acid mediated signaling pathway” led to more detailed analysis of gibberellin metabolism during the reproductive transition and proved to be significant. Fewer terms were enriched between 1MI SAM tissue and S SAM tissue consistent with a return to normal SAM activity as the inflorescence grows and extends prior to the development of umbels.

**Fig. 9:**
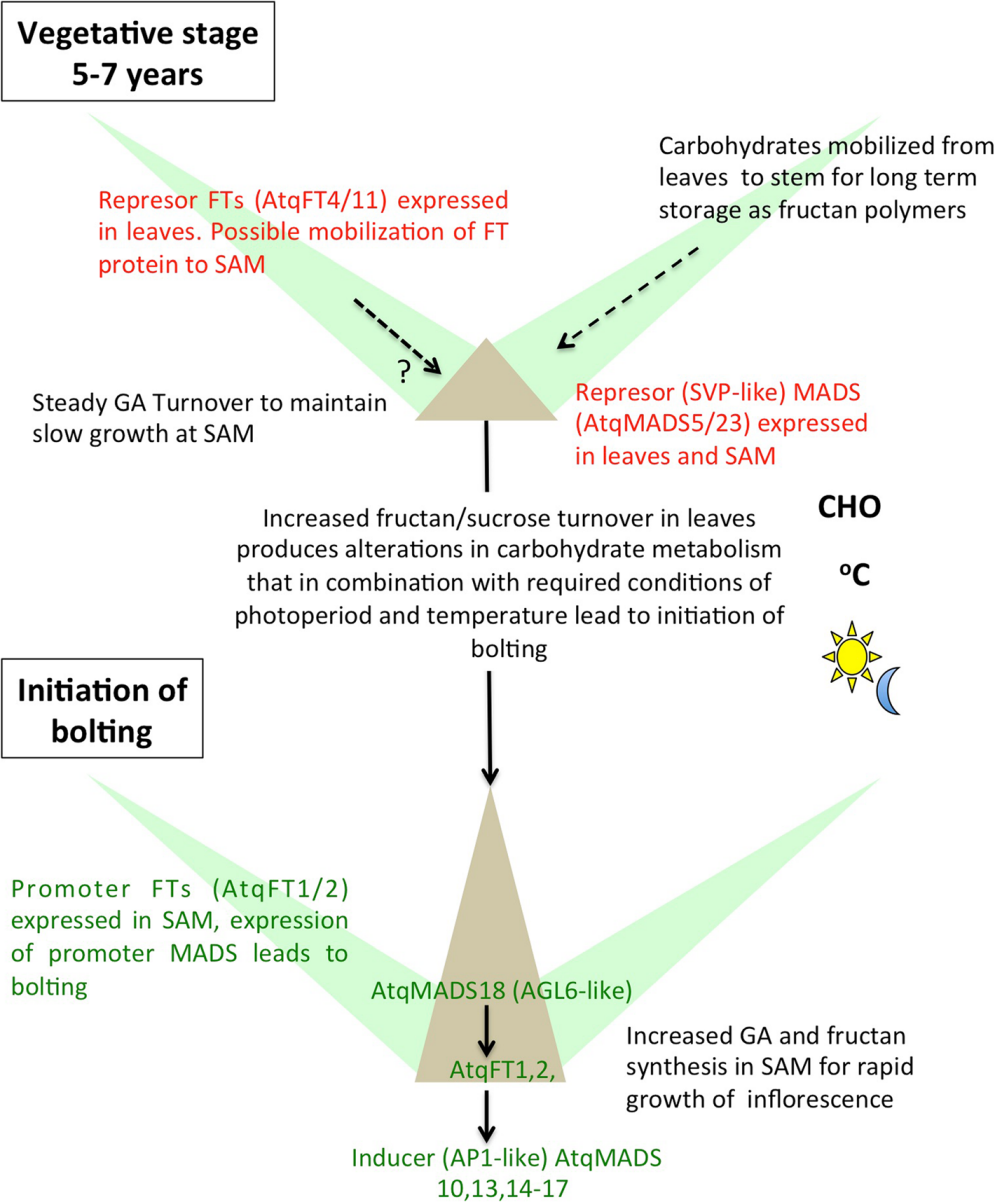
Model incorporating factors involved in the initiation of bolting in *A. tequilana*

To explore the putative roles of specific genes and types of metabolism in relation to bolting in *A. tequilana*, in silico expression patterns for the Flowering Locus Time (FT) and MADS box gene families, and genes involved in fructan and gibberellin metabolism were analyzed in detail. Fifteen distinct members of the FT gene family could be identified for *A. tequilana* and in other plant species, genes classified as “true” FT type have been shown to regulate the bolting process by acting as promoters or repressors [10, 11]. Expression patterns for the *A. tequilana* genes sharing the same predicted function (repressor or promoter) were consistent, suggesting that AtqFT 11 and 4 could act as repressors active in leaves and be putatively transported to the SAM to repress other (promoter) FTs (for example AtqFT 10 and 7). As the transition process is initiated AtqFT 8,9 and 6 become expressed in leaves and AtqFT 1 and 2 in leaves and SAM. The flowering promoter type FT genes expressed in leaves could also be transported to the SAM and interact and/or interfere with the AtqFT4 and 11 repressors leading to the expression of AtqFT 10 and 7 or could directly stimulate the expression of these genes, thereby initiating bolting. The complexity of the system would permit the response to different environmental and endogenous stimuli, each affecting and regulating specific FT genes and only when all internal and external conditions were met and the correct combination of FT proteins achieved would the signals converge to produce the transition (Fig. 9). This hypothesis accounts for the particular case of bolting in perennial monocarpic species and is supported by the qRT-PCR results.

Another key gene family in the transition to flowering is the MADS box family. This report extends the number of reported *A. tequilana* MADS box type II genes from 7 to 25. Four of the genes reported previously were not identified probably because floral organs such as tepals, anthers and ovaries were omitted. Although expression patterns consistent with the roles of Apetala and SVP type MADS were observed, the expression patterns for the *A. tequilana* SOC1 type MADS differ significantly from the expected based on data from *A. thaliana*. However in rice, OsMADS 50 and OsMADS 56 (closely related to AtqMADS24 and AtqMADS25) have been shown to physically interact and have antagonistic functions [45]. In rice expression of both genes in leaf tissue represses the activation of the rice FT-like protein, Hd3a/RFT1, thereby controlling the transition to flowering and a similar mechanism could therefore regulate this process in *A. tequilana*. The expression pattern of AtqMADS18 is consistent with the FT promoting role previously described for the *A. thaliana* AGL6 gene [46], suggesting that AtqMADS18 may have a similar function.

Analysis of in silico expression patterns for genes encoding enzymes involved in fructan metabolism uncovered very clear patterns associated with tissue type. Interestingly, genes encoding enzymes involved in fructan synthesis were strongly expressed in SAM tissue but not in leaves whereas those encoding fructan-degrading enzymes showed the opposite pattern. Furthermore, Atq6G1FFT2 is specifically expressed in S SAM tissue and to a lesser extent in S leaves. The 6GFFT enzymes produce “neo” type fructans [47] and it could be speculated that specific types of fructan polymers could serve as signals at particular developmental stages. Another intriguing result is the pattern of expression of the gene denominated AtqInv2 which has not been clearly classified as an invertase or a fructosyl transferase [20] based on sequence data or in vitro activity assays (data not shown). It will be interesting therefore to determine the functional role of the AtqInv2 in future studies.

Gibberellin metabolism genes showed clear patterns of differential expression not only between leaves and meristems but also between meristem tissues at different developmental stages. Genes encoding both synthetic and deactivating enzymes for gibberellin metabolism are expressed in vegetative SAM, indicating that GA synthesis must be regulated to maintain the active growth of the V SAM. In contrast genes encoding deactivating enzymes are down regulated in both S and 1MI stages, suggesting that gibberellin plays an essential role in process of bolting.

## Conclusions

RNAseq analysis led to the development of an efficient strategy for transcriptome development, assembly and analysis in *Agave* species and uncovered evidence for the presence at significant levels of incompletely processed transcripts that may play a role in transcript regulation or affect the functions of encoded proteins.

GO enrichment and in silico expression analysis data can be summarized in a preliminary model (Fig. 9) that describes the putative roles of FT and MADS box proteins, gibberellins and carbohydrates in the determination of the initiation of bolting in *A. tequilana*. The data suggest that at the vegetative stage, repressor type FT isoforms (AtqFT4 and 11) are expressed in leaves and could putatively be transported through the phloem to the SAM (a definition of florigen factors) where they would interact with other (promoter) FT isoforms to inhibit the transition to reproductive development. A specific combination of environmental (photoperiod, temperature) and endogenous (carbohydrates, GA) factors potentially converge on distinct FT proteins to release the FT imposed inhibition of bolting, permitting the transformation of the SAM to an inflorescence meristem by the activation of specific MADS box proteins. Although undoubtedly the transition process is more complex, this model provides a framework from which to begin to study it in more detail at the molecular and functional level in *Agave* species.

## Additional files

Additional file 1: Stages of bolting and examples of SAM tissue in *A. tequilana*. (ZIP 549 kb)

Additional file 2: Comparison of BUSCO analysis for transcriptomes generated by Trinity 2.2.0 *kmer* 31, Oases 0.2.08 *kmer* 31 a 77 and Gross et al. 2013. (JPG 180 kb)

Additional file 3: Expression pattern of Atq1-SST2 contigs. (JPG 211 kb)

Additional file 4: Summary of percentages and GC content of different types of sequences identified in the *A. tequilana* transcriptome. (JPG 224 kb)

Additional file 5: Heat map of differentially expressed genes in leaves and SAM at different developmental stages. (JPG 547 kb)

Additional file 6: Enrichment of transcripts overexpressed in leaves at vegetative (V) and sunken (S) stages. (JPG 259 kb)

Additional file 7: Enrichment of transcripts overexpressed in SAM vegetative (V) and sunken (S) stages. (JPG 479 kb)

Additional file 8: Enrichment of transcripts overexpressed in Leaves at sunken (S) and 1 m inflorescence (1MI) stages. (JPG 448 kb)

Additional file 9: Enrichment of transcripts overexpressed in SAM at sunken (S) and 1 m inflorescence (1MI) stages. (JPG 454 kb)

Additional file 10: List of *A. tequilana* FT, MADS and Gibberellin metabolism genes and GenBank accession numbers. (JPG 391 kb)

Additional file 11: Motifs that determine repressor or inducer activity of FT proteins. (JPG 711 kb)

Additional file 12: Identification of Class I and Class II *A. tequilana* MADS box genes. (JPG 393 kb)

## Data Availability

Sequences for all characterized cDNAs have been deposited in GenBank and accession numbers are listed in Additional file 10. Raw sequence data generated in this work are available through the NCBI Sequence Read Archive (SRA), study accession PRJNA507288.

## Abbreviations

CPAT: Coding-Potential Assessment Tool
CPC: Coding Protein Calculator
DP: Degree of polymerization
FDR: False Discovery Rate
FT: Flowering locus T
GA: Gibberellin
GO: Gene Ontology
I1M: 1 m inflorescence
PEBP: phosphatidylethanolamine binding protein
RdP: Real de Penjamo
S: initiation of bolting
SAM: Shoot apical meristem
TS: Tequila Sauza
V: vegetative

## References

[1] Dutta S, Biswas P, Chakraborty S, Mitra D, Pal A, Das M (2018). Identification, characterization and gene expression analyses of important flowering genes related to photoperiodic pathway in bamboo. BMC Genomics.

[2] Valenzuela A (2011). A new agenda for blue agave landraces: food, energy and tequila. GCB Bioenergy.

[3] Valenzuela A: El agave tequilero: su cultivo e industria de México: Mundiprensa Editores. México; 2003.

[4] Debnath M, Pandey M, Sharma R, Thakur GS, Lal P (2010). Biotechnological intervention of Agave sisalana: a unique fiber yielding plant with medicinal property. Journal of Medicinal Plants Research.

[5] Huang X, Wang B, Xi J, Zhang Y, He C, Zheng J, Gao J, Chen H, Zhang S, Wu W (2018). Transcriptome comparison reveals distinct selection patterns in domesticated and wild Agave species, the important CAM plants. Int J Genomics.

[6] Yang X, Cushman JC, Borland AM, Edwards EJ, Wullschleger SD, Tuskan GA, Owen NA, Griffiths H, Smith JA, De Paoli HC (2015). A roadmap for research on crassulacean acid metabolism (CAM) to enhance sustainable food and bioenergy production in a hotter, drier world. New Phytol.

[7] Cushman JC, Davis SC, Yang X, Borland AM. Development and use of bioenergy feedstocks for semi-arid and arid lands. J Exp Bot. 2015. 10.1093/jxb/erv087 25873672

[8] Delgado Sandoval Sdel C, Abraham Juarez MJ, Simpson J (2012). Agave tequilana MADS genes show novel expression patterns in meristems, developing bulbils and floral organs. Sex Plant Reprod.

[9] Khan MR, Ai XY, Zhang JZ (2014). Genetic regulation of flowering time in annual and perennial plants. Wiley Interdiscip Rev RNA.

[10] Karlgren A, Gyllenstrand N, Kallman T, Sundstrom JF, Moore D, Lascoux M, Lagercrantz U (2011). Evolution of the PEBP gene family in plants: functional diversification in seed plant evolution. Plant Physiol.

[11] Pin PA, Benlloch R, Bonnet D, Wremerth-Weich E, Kraft T, Gielen JJ, Nilsson O (2010). An antagonistic pair of FT homologs mediates the control of flowering time in sugar beet. Science.

[12] Hsu CY, Adams JP, Kim H, No K, Ma C, Strauss SH, Drnevich J, Vandervelde L, Ellis JD, Rice BM (2011). FLOWERING LOCUS T duplication coordinates reproductive and vegetative growth in perennial poplar. Proc Natl Acad Sci U S A.

[13] Hsu CY, Liu Y, Luthe DS, Yuceer C (2006). Poplar FT2 shortens the juvenile phase and promotes seasonal flowering. Plant Cell.

[14] King RW, Moritz T, Evans LT, Martin J, Andersen CH, Blundell C, Kardailsky I, Chandler PM (2006). Regulation of flowering in the long-day grass Lolium temulentum by gibberellins and the FLOWERING LOCUS T gene. Plant Physiol.

[15] Wilkie JD, Sedgley M, Olesen T (2008). Regulation of floral initiation in horticultural trees. J Exp Bot.

[16] Munoz-Fambuena N, Mesejo C, Gonzalez-Mas MC, Primo-Millo E, Agusti M, Iglesias DJ (2012). Fruit load modulates flowering-related gene expression in buds of alternate-bearing ‘Moncada’ mandarin. Ann Bot.

[17] Munoz-Fambuena N, Mesejo C, Gonzalez-Mas MC, Primo-Millo E, Agusti M, Iglesias DJ (2011). Fruit regulates seasonal expression of flowering genes in alternate-bearing ‘Moncada’ mandarin. Ann Bot.

[18] Li J, Pan B-Z, Niu L, Chen M-S, Tang M, Xu Z-F (2018). Gibberellin inhibits floral initiation in the perennial Woody Plant Jatropha curcas. J Plant Growth Regul.

[19] Boss PK, Thomas MR (2002). Association of dwarfism and floral induction with a grape ‘green revolution’ mutation. Nature.

[20] Avila de Dios E, Gomez Vargas AD, Damian Santos ML, Simpson J (2015). New insights into plant glycoside hydrolase family 32 in Agave species. Front Plant Sci.

[21] Bolger AM, Lohse M, Usadel B (2014). Trimmomatic: a flexible trimmer for Illumina sequence data. Bioinformatics.

[22] Zhao QY, Wang Y, Kong YM, Luo D, Li X, Hao P (2011). Optimizing de novo transcriptome assembly from short-read RNA-Seq data: a comparative study. BMC bioinformatics.

[23] Schulz MH, Zerbino DR, Vingron M, Birney E (2012). Oases: robust de novo RNA-seq assembly across the dynamic range of expression levels. Bioinformatics.

[24] Zerbino DR: Using the velvet de novo assembler for short-read sequencing technologies. Curr Protoc Bioinformatics 2010, Chapter 11:Unit 11 15. 10.1002/0471250953.bi1105s31 PMC2952100 20836074

[25] Zerbino DR, Birney E (2008). Velvet: algorithms for de novo short read assembly using de Bruijn graphs. Genome Res.

[26] Fu L, Niu B, Zhu Z, Wu S, Li W (2012). CD-HIT: accelerated for clustering the next-generation sequencing data. Bioinformatics.

[27] Huang Y, Niu B, Gao Y, Fu L, Li W (2010). CD-HIT suite: a web server for clustering and comparing biological sequences. Bioinformatics.

[28] Wang L, Park HJ, Dasari S, Wang S, Kocher JP, Li W (2013). CPAT: coding-potential assessment tool using an alignment-free logistic regression model. Nucleic Acids Res.

[29] Kong L, Zhang Y, Ye ZQ, Liu XQ, Zhao SQ, Wei L, Gao G (2007). CPC: assess the protein-coding potential of transcripts using sequence features and support vector machine. Nucleic Acids Res.

[30] Robinson MD, McCarthy DJ, Smyth GK (2010). edgeR: a Bioconductor package for differential expression analysis of digital gene expression data. Bioinformatics.

[31] Du Z, Zhou X, Ling Y, Zhang Z, Su Z (2010). agriGO: a GO analysis toolkit for the agricultural community. Nucleic Acids Res.

[32] Supek F, Bosnjak M, Skunca N, Smuc T (2011). REVIGO summarizes and visualizes long lists of gene ontology terms. PLoS One.

[33] Lopez MG, Mancilla-Margalli NA, Mendoza-Diaz G (2003). Molecular structures of fructans from Agave tequilana Weber var. Azul. J Agric Food Chem.

[34] Mancilla-Margalli NA, Lopez MG (2006). Water-soluble carbohydrates and fructan structure patterns from Agave and Dasylirion species. J Agric Food Chem.

[35] Mellado-Mojica E, Lopez MG (2012). Fructan metabolism in a. tequilana Weber blue variety along its developmental cycle in the field. J Agric Food Chem.

[36] Gross SM, Martin JA, Simpson J, Abraham-Juarez MJ, Wang Z, Visel A (2013). De novo transcriptome assembly of drought tolerant CAM plants, Agave deserti and Agave tequilana. BMC Genomics.

[37] Cortes-Romero C, Martinez-Hernandez A, Mellado-Mojica E, Lopez MG, Simpson J (2012). Molecular and functional characterization of novel fructosyltransferases and invertases from Agave tequilana. PLoS One.

[38] Zavala-García LE, Sánchez-Segura L, de Dios EA, Pérez-López A, Simpson J (2018). Starch accumulation is associated with active growth in a. tequilana. Plant Physiol Biochem.

[39] Yang L, Xu M, Koo Y, He J, Poethig RS (2013). Sugar promotes vegetative phase change in Arabidopsis thaliana by repressing the expression of MIR156A and MIR156C. Elife.

[40] Yu S, Cao L, Zhou CM, Zhang TQ, Lian H, Sun Y, Wu J, Huang J, Wang G, Wang JW (2013). Sugar is an endogenous cue for juvenile-to-adult phase transition in plants. Elife.

[41] Yin H, Guo HB, Weston DJ, Borland AM, Ranjan P, Abraham PE, Jawdy SS, Wachira J, Tuskan GA, Tschaplinski TJ (2018). Diel rewiring and positive selection of ancient plant proteins enabled evolution of CAM photosynthesis in Agave. BMC Genomics.

[42] Cervantes-Perez SA, Espinal-Centeno A, Oropeza-Aburto A, Caballero-Perez J, Falcon F, Aragon-Raygoza A, Sanchez-Segura L, Herrera-Estrella L, Cruz-Hernandez A, Cruz-Ramirez A (2018). Transcriptional profiling of the CAM plant Agave salmiana reveals conservation of a genetic program for regeneration. Dev Biol.

[43] McKain MR, Wickett N, Zhang Y, Ayyampalayam S, McCombie WR, Chase MW, Pires JC, dePamphilis CW, Leebens-Mack J (2012). Phylogenomic analysis of transcriptome data elucidates co-occurrence of a paleopolyploid event and the origin of bimodal karyotypes in Agavoideae (Asparagaceae). Am J Bot.

[44] Bournay AS, Hedley PE, Maddison A, Waugh R, Machray GC (1996). Exon skipping induced by cold stress in a potato invertase gene transcript. Nucleic Acids Res.

[45] Ryu CH, Lee S, Cho LH, Kim SL, Lee YS, Choi SC, Jeong HJ, Yi J, Park SJ, Han CD (2009). OsMADS50 and OsMADS56 function antagonistically in regulating long day (LD)-dependent flowering in rice. Plant Cell Environ.

[46] Yoo SK, Wu X, Lee JS, Ahn JH (2011). AGAMOUS-LIKE 6 is a floral promoter that negatively regulates the FLC/MAF clade genes and positively regulates FT in Arabidopsis. Plant J.

[47] Van den Ende W, Lammens W, Van Laere A, Schroeven L, Le Roy K (2009). Donor and acceptor substrate selectivity among plant glycoside hydrolase family 32 enzymes. FEBS J.

