# Supplementary figures and images for: Transcriptome analysis of bolting in A. tequilana reveals roles for florigen, MADS, fructans and gibberellins
Source: BMC Genomics. 2019 Jun 10;20:473. doi: 10.1186/s12864-019-5808-9 (PMC6558708; doi:10.1186/s12864-019-5808-9)

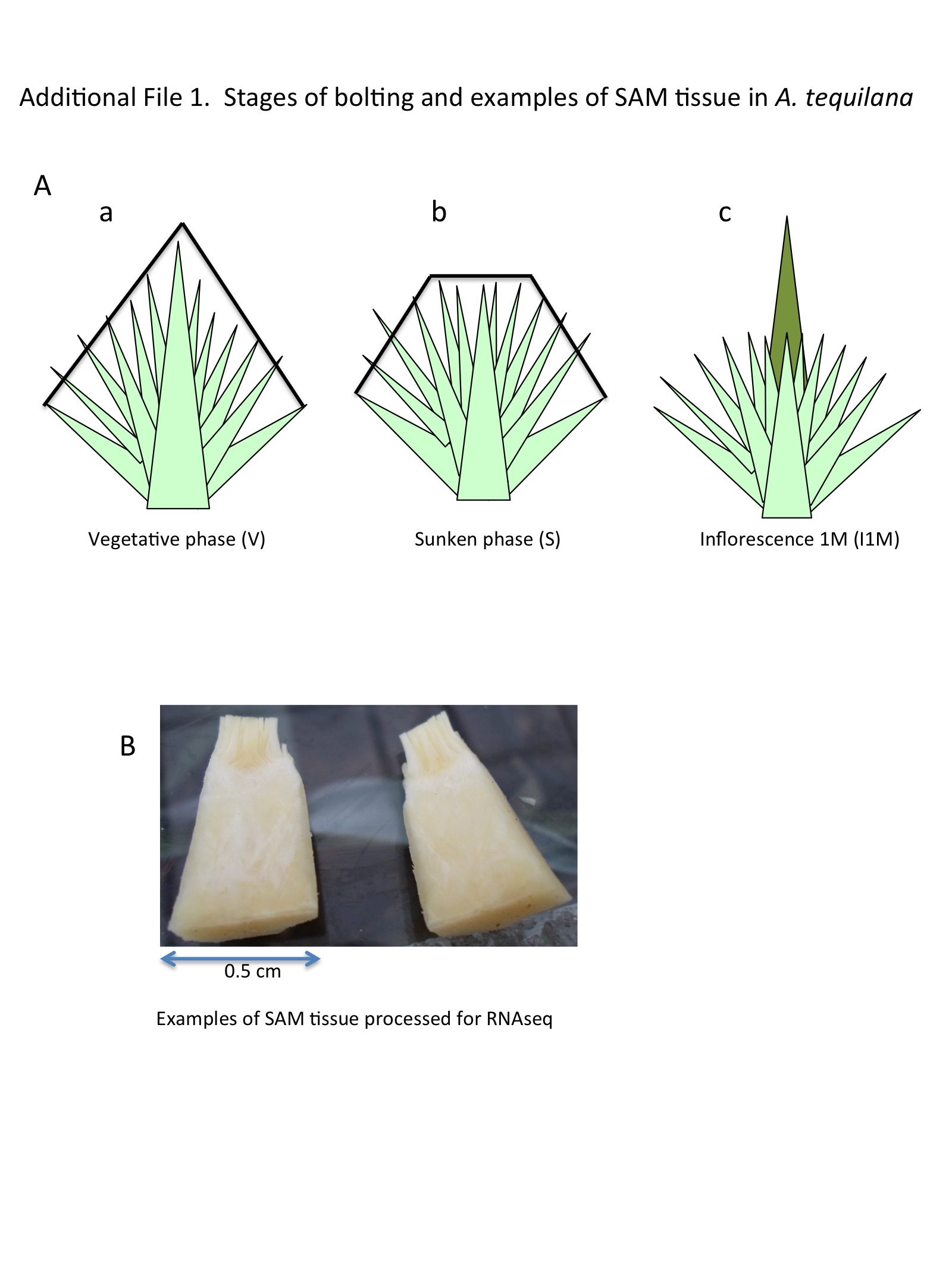

Supplement: Supplementary file 1 — Stages of bolting and examples of SAM tissue in A. tequilana. (ZIP 549 kb) [file 12864_2019_5808_MOESM1_ESM.zip › Additional file1v2.jpg]

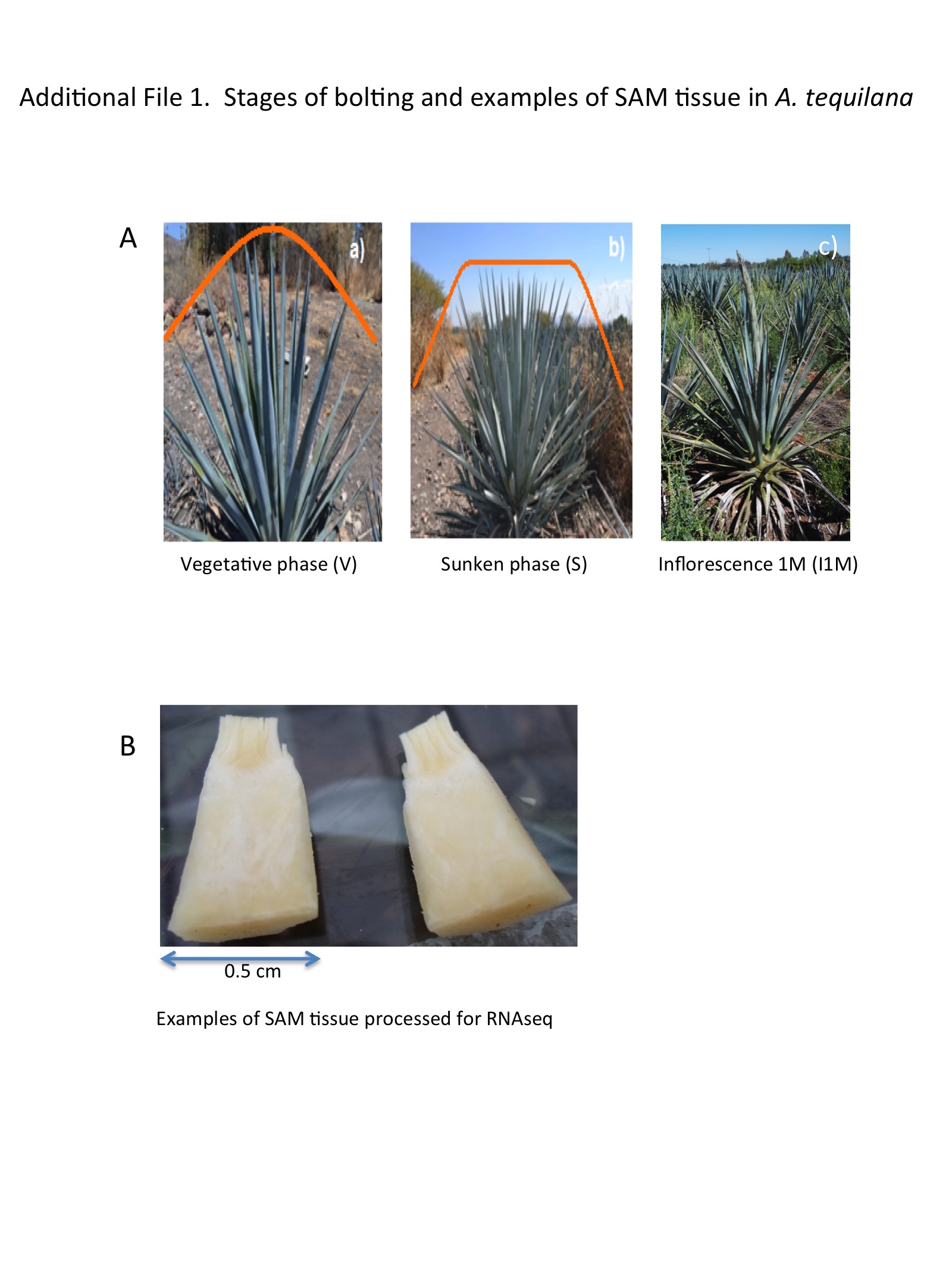

Supplement: Supplementary file 1 — Stages of bolting and examples of SAM tissue in A. tequilana. (ZIP 549 kb) [file 12864_2019_5808_MOESM1_ESM.zip › Slide01.jpg]

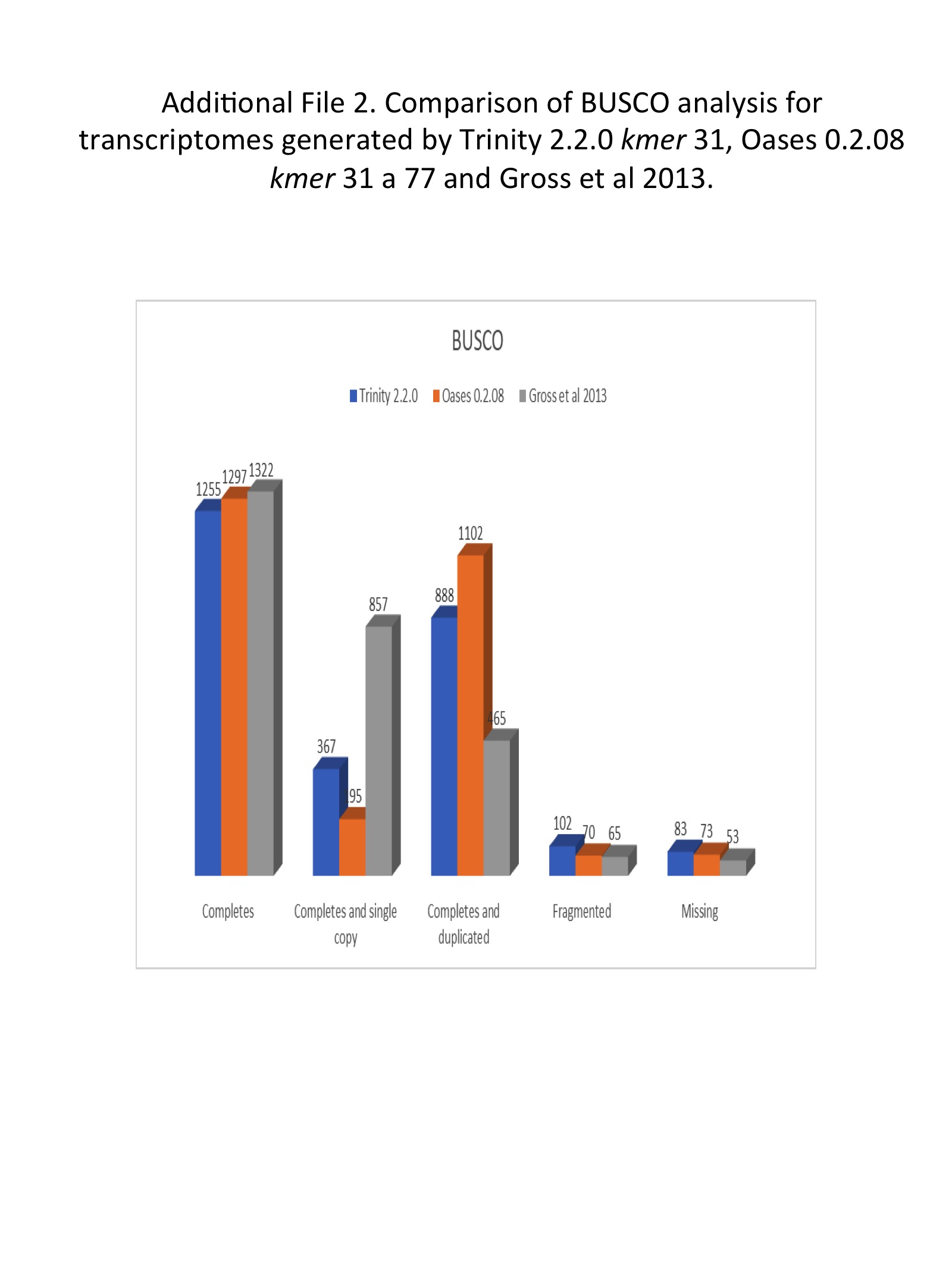

Supplement: Supplementary file 2 — Comparison of BUSCO analysis for transcriptomes generated by Trinity 2.2.0 kmer 31, Oases 0.2.08 kmer 31 a 77 and Gross et al. 2013. (JPG 180 kb) [file 12864_2019_5808_MOESM2_ESM.jpg]

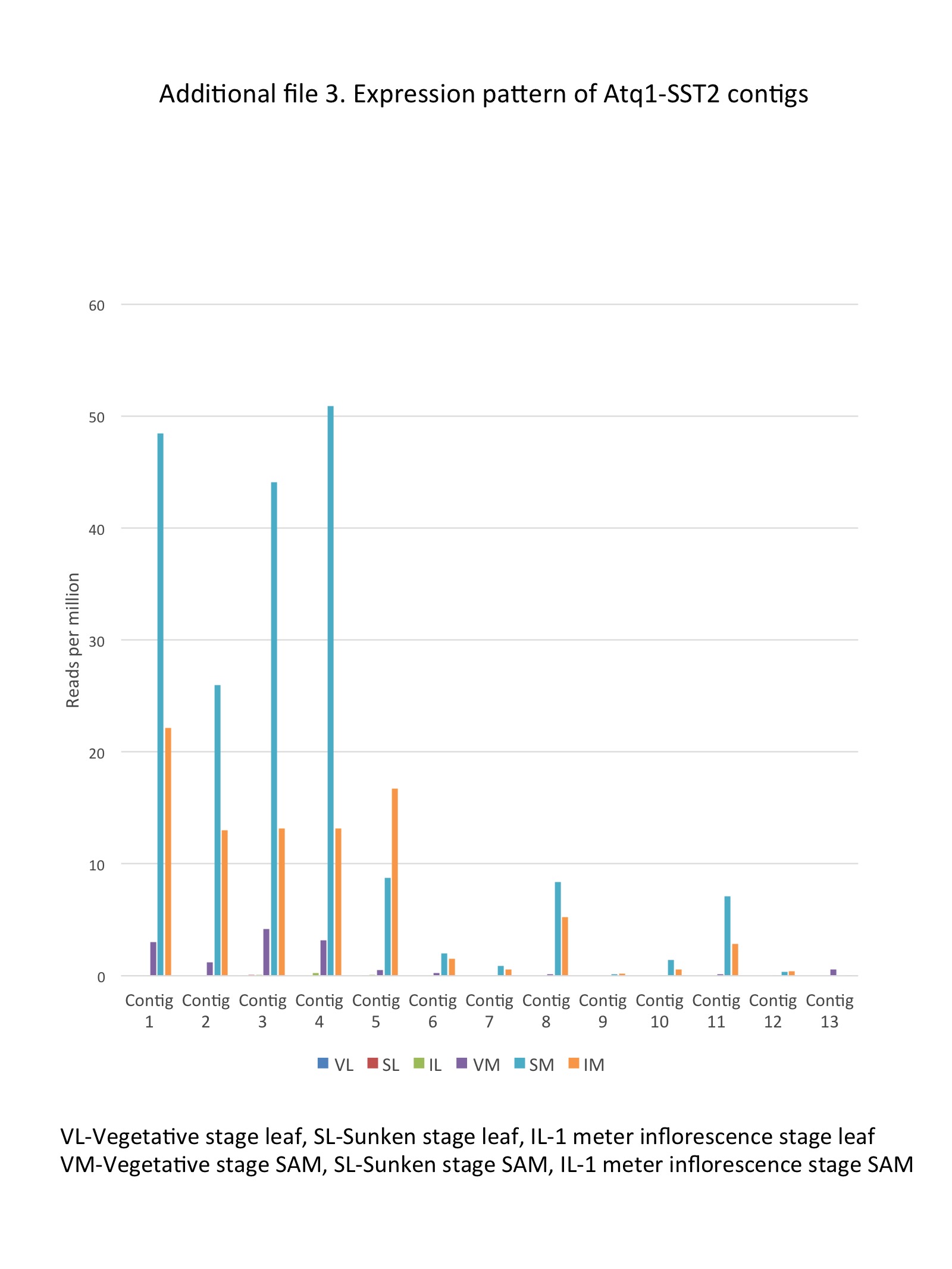

Supplement: Supplementary file 3 — Expression pattern of Atq1-SST2 contigs. (JPG 211 kb) [file 12864_2019_5808_MOESM3_ESM.jpg]

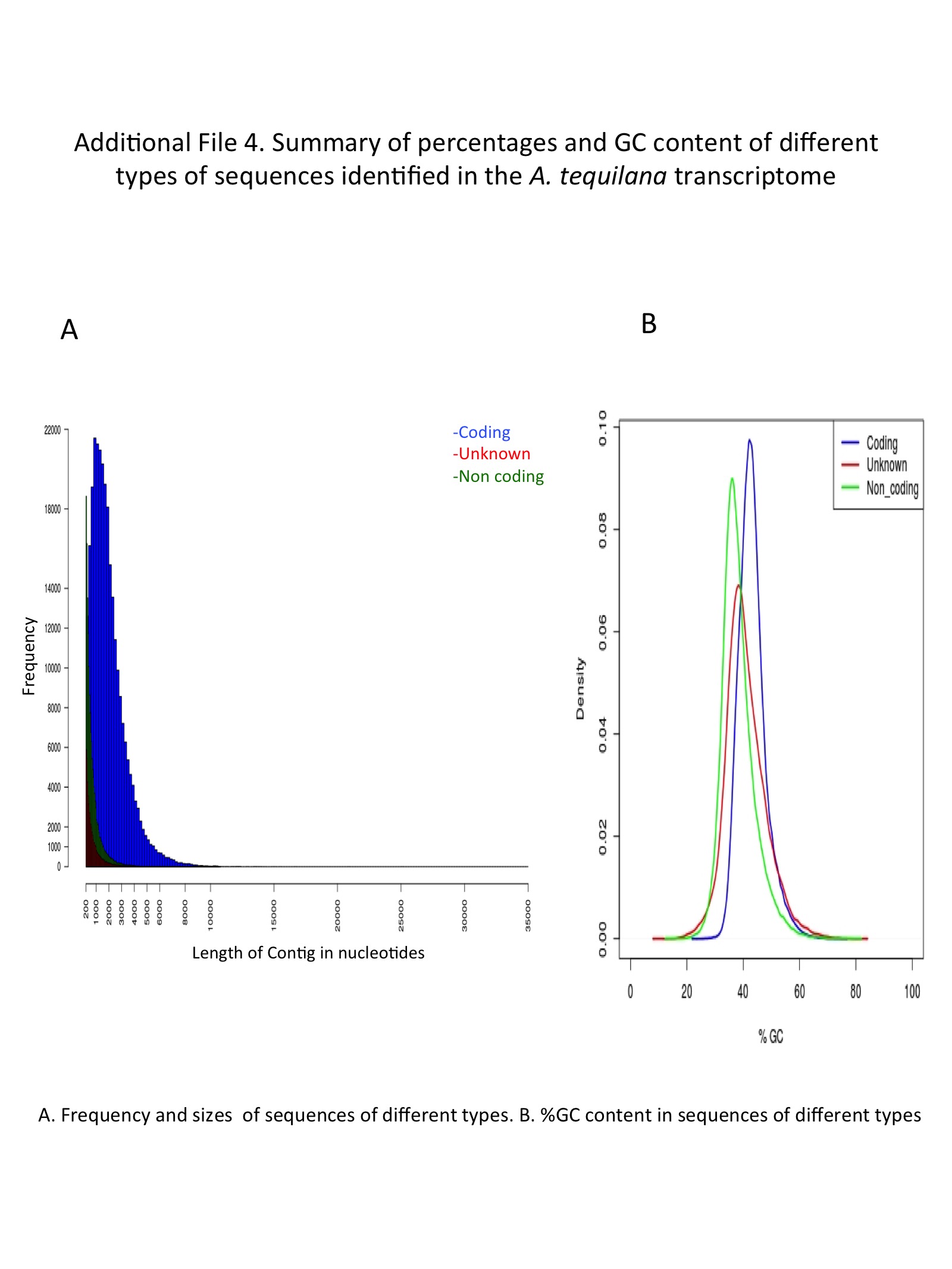

Supplement: Supplementary file 4 — Summary of percentages and GC content of different types of sequences identified in the A. tequilana transcriptome. (JPG 224 kb) [file 12864_2019_5808_MOESM4_ESM.jpg]

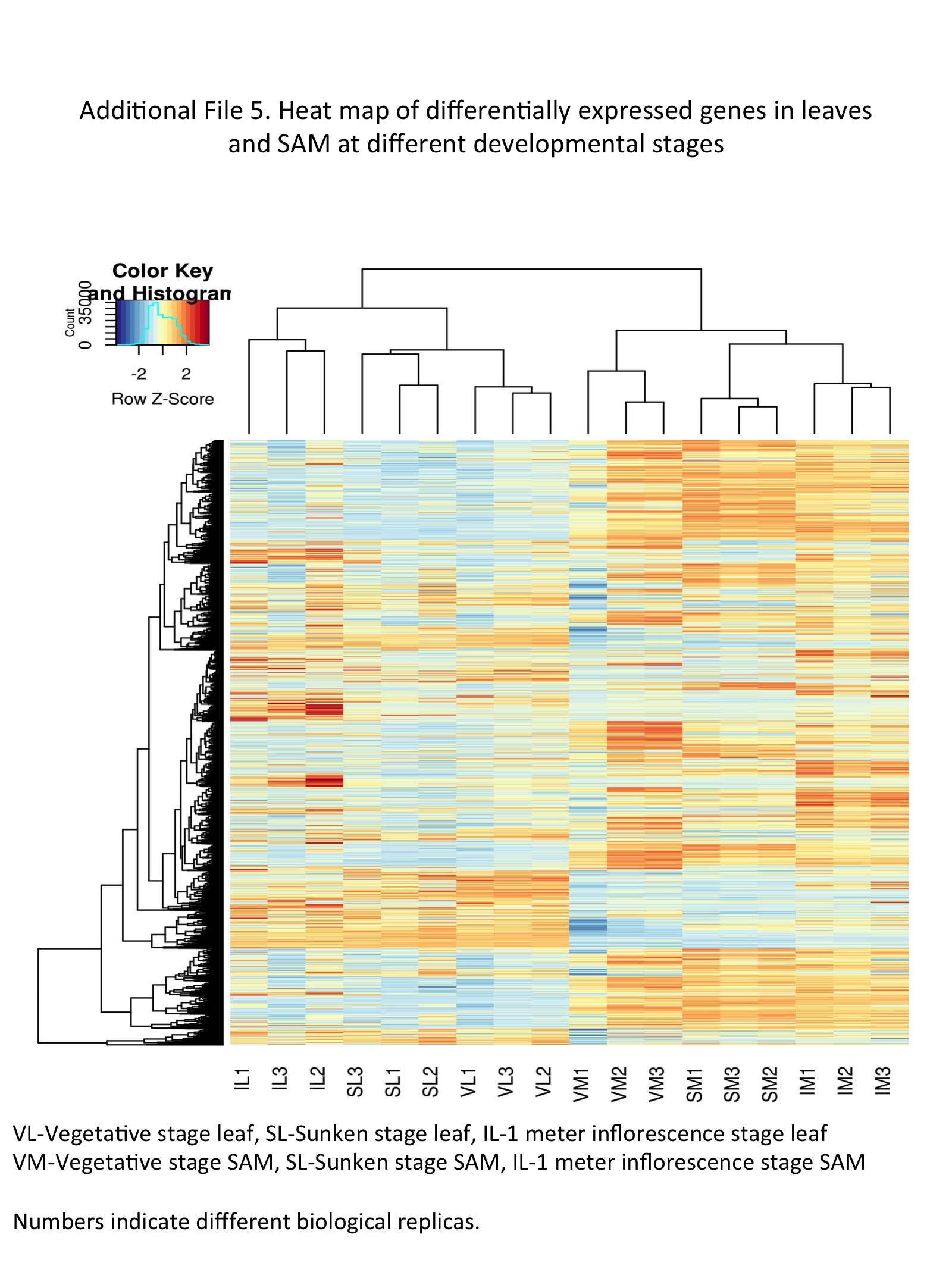

Supplement: Supplementary file 5 — Heat map of differentially expressed genes in leaves and SAM at different developmental stages. (JPG 547 kb) [file 12864_2019_5808_MOESM5_ESM.jpg]

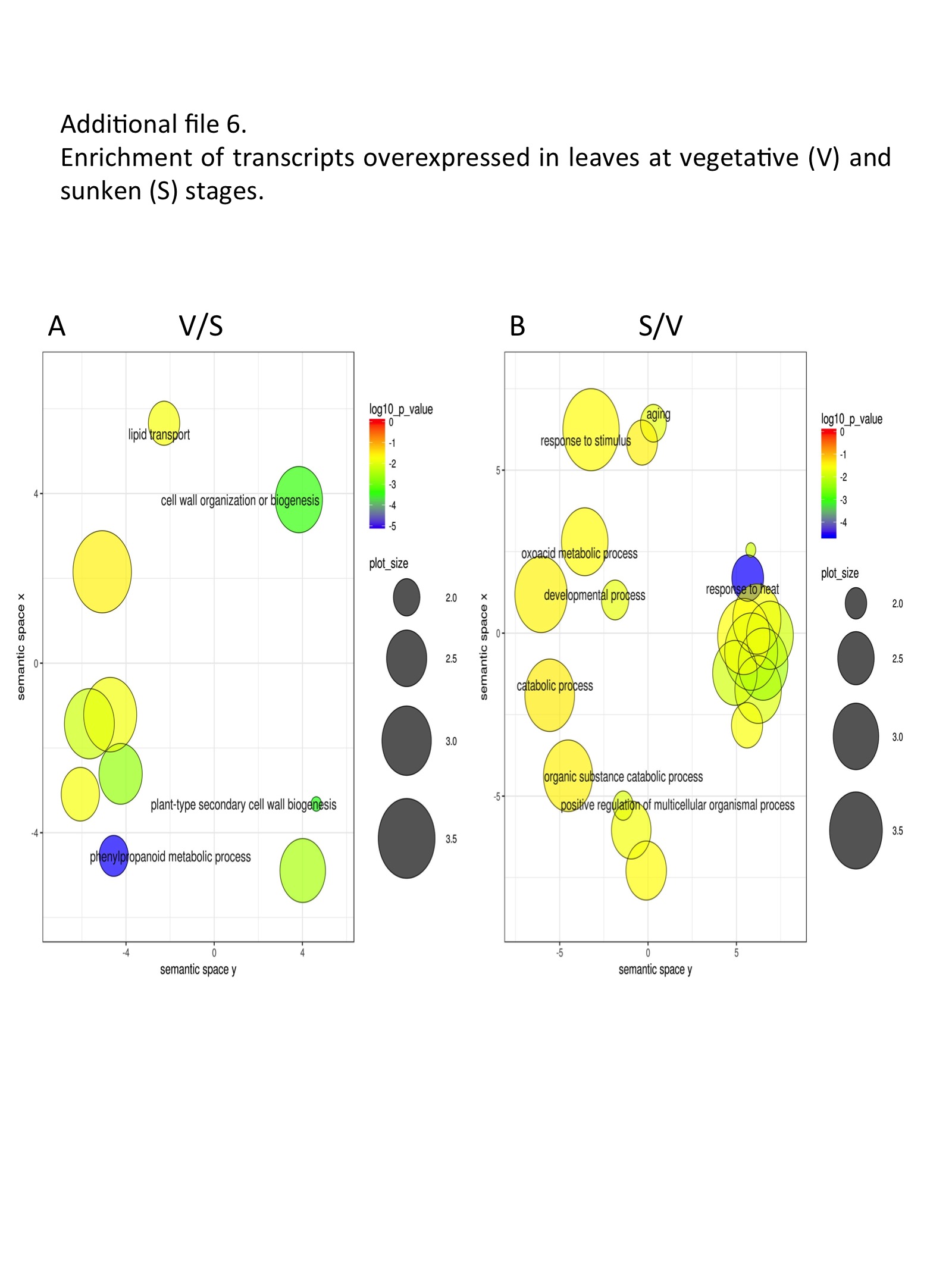

Supplement: Supplementary file 6 — Enrichment of transcripts overexpressed in leaves at vegetative (V) and sunken (S) stages. (JPG 259 kb) [file 12864_2019_5808_MOESM6_ESM.jpg]

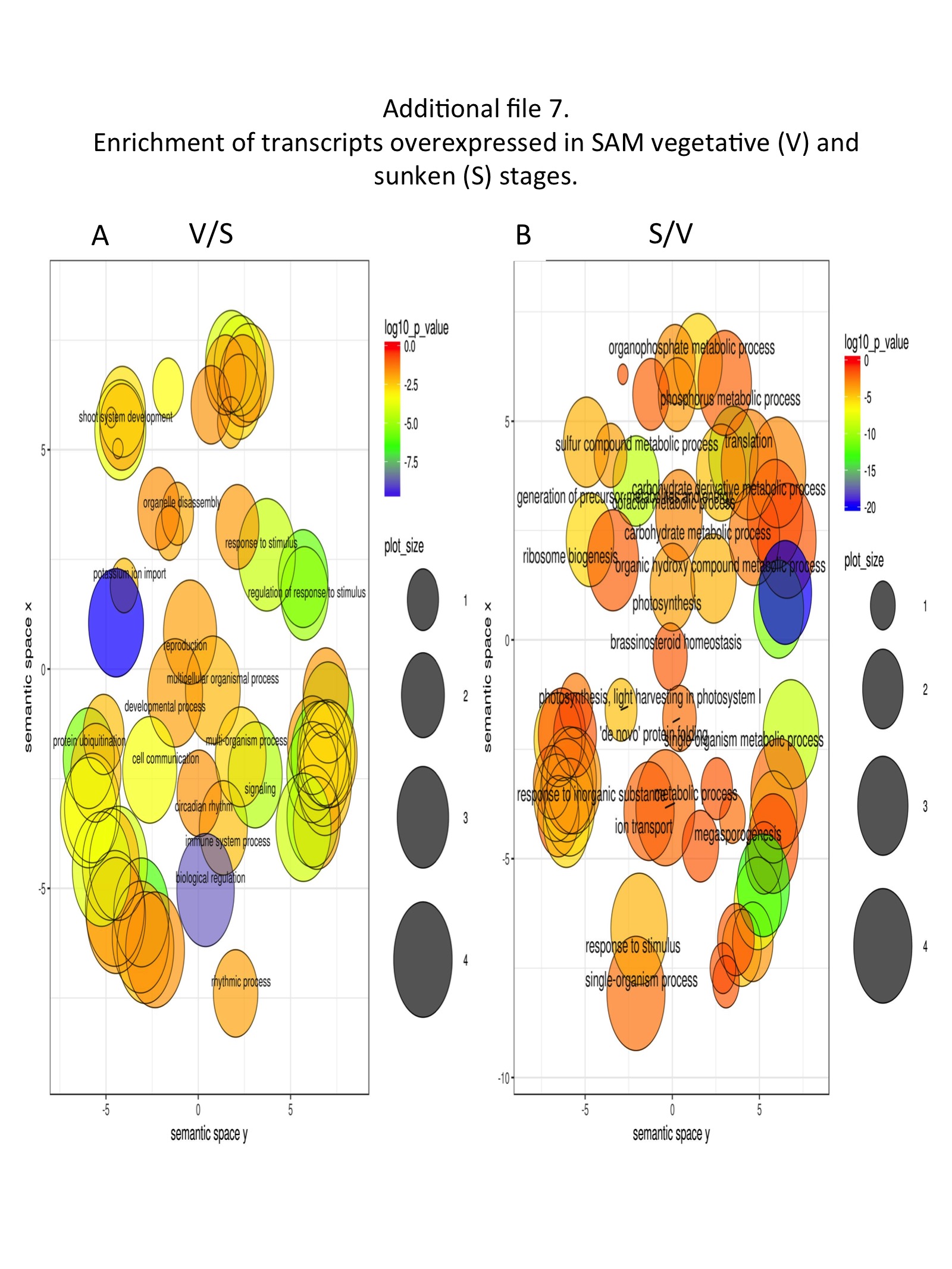

Supplement: Supplementary file 7 — Enrichment of transcripts overexpressed in SAM vegetative (V) and sunken (S) stages. (JPG 479 kb) [file 12864_2019_5808_MOESM7_ESM.jpg]

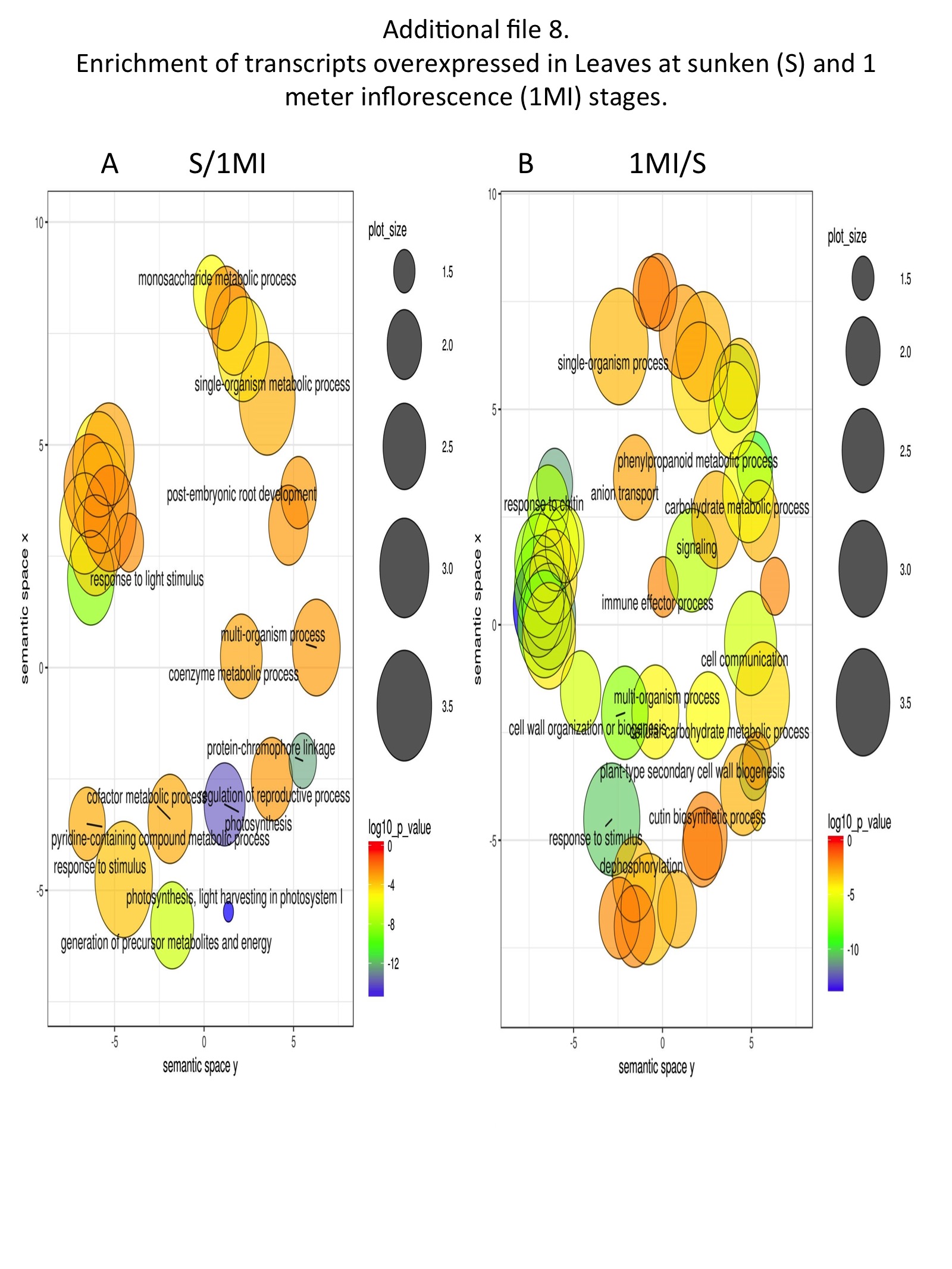

Supplement: Supplementary file 8 — Enrichment of transcripts overexpressed in Leaves at sunken (S) and 1 m inflorescence (1MI) stages. (JPG 448 kb) [file 12864_2019_5808_MOESM8_ESM.jpg]

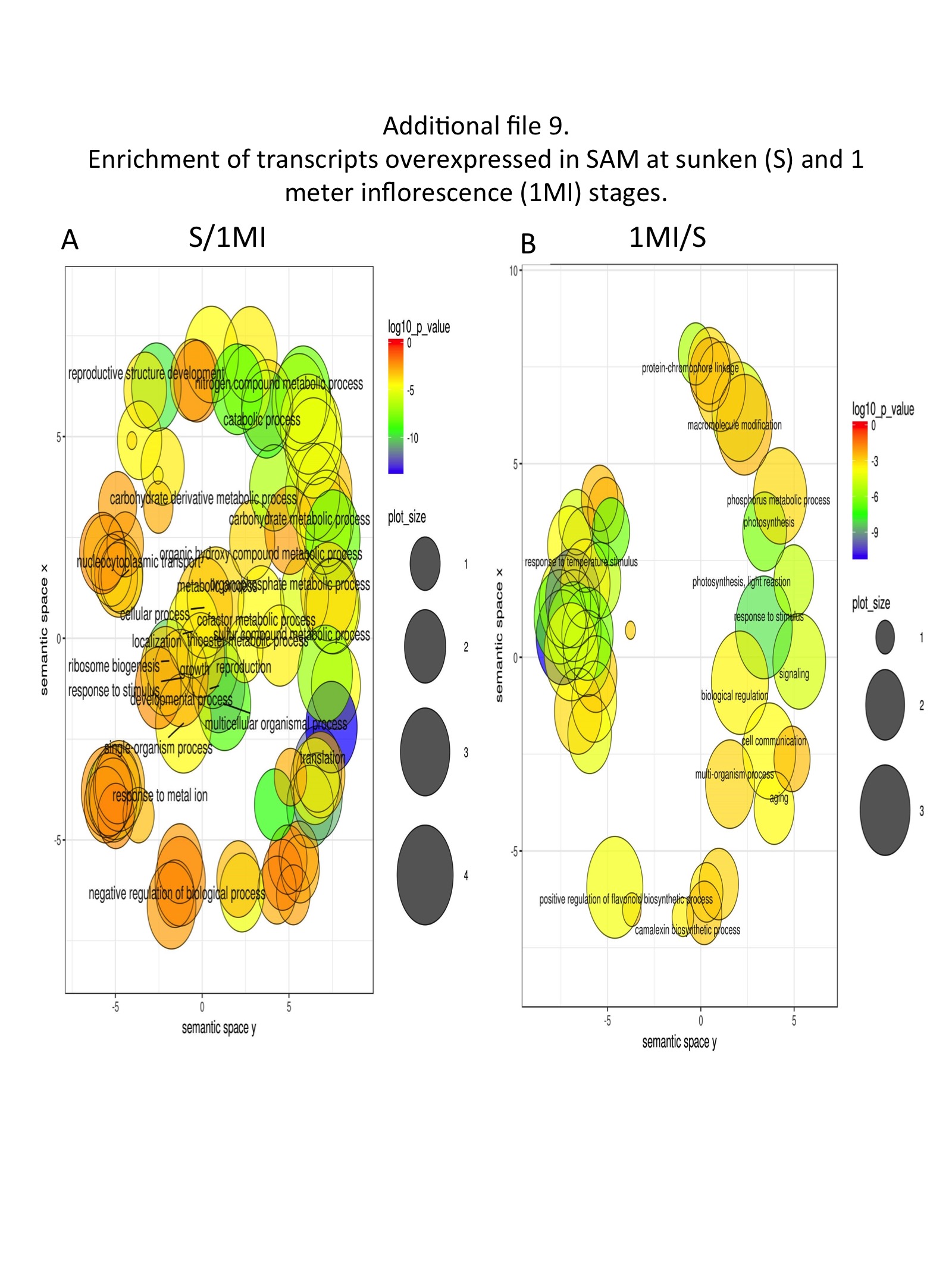

Supplement: Supplementary file 9 — Enrichment of transcripts overexpressed in SAM at sunken (S) and 1 m inflorescence (1MI) stages. (JPG 454 kb) [file 12864_2019_5808_MOESM9_ESM.jpg]

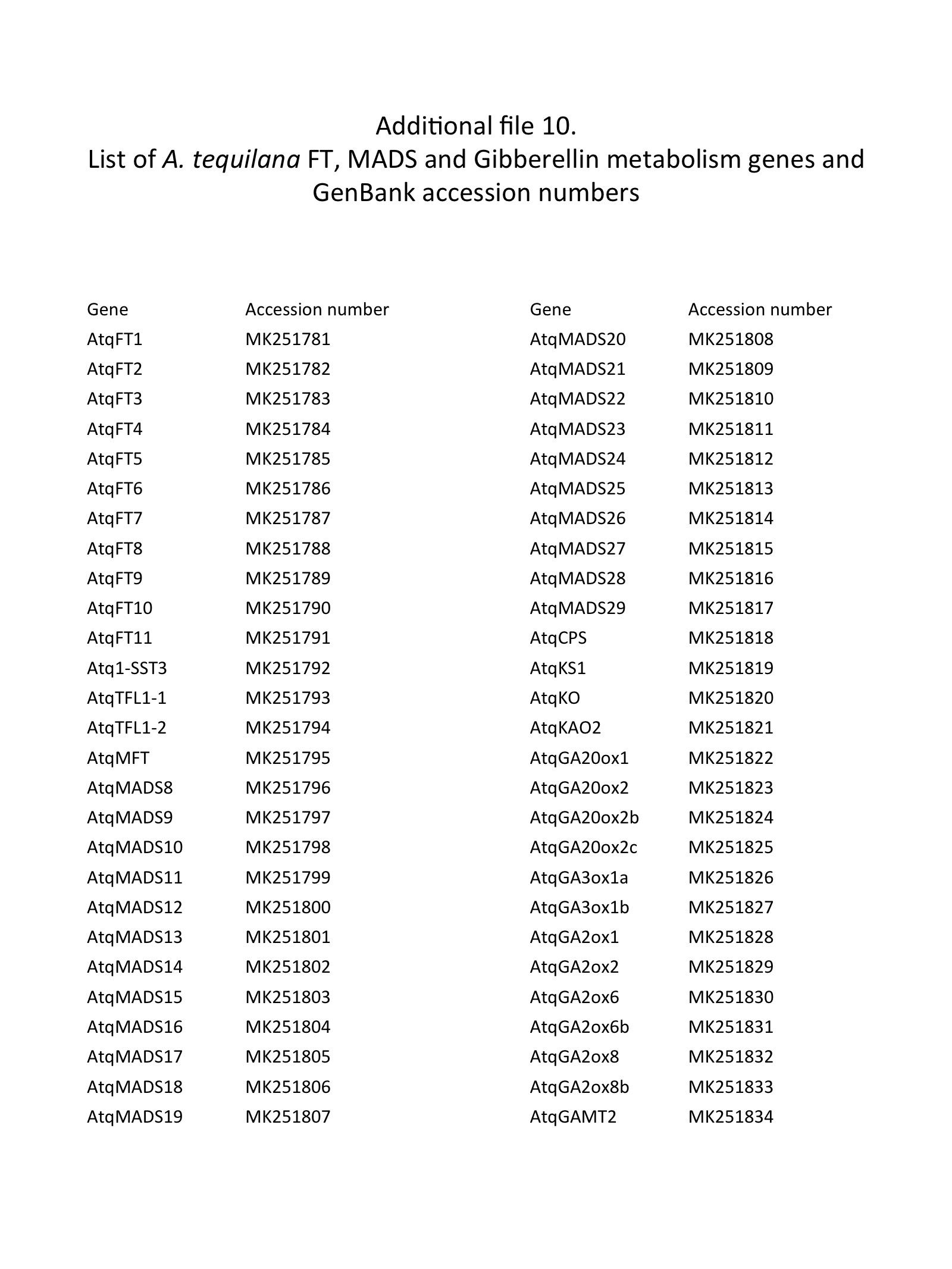

Supplement: Supplementary file 10 — List of A. tequilana FT, MADS and Gibberellin metabolism genes and GenBank accession numbers. (JPG 391 kb) [file 12864_2019_5808_MOESM10_ESM.jpg]

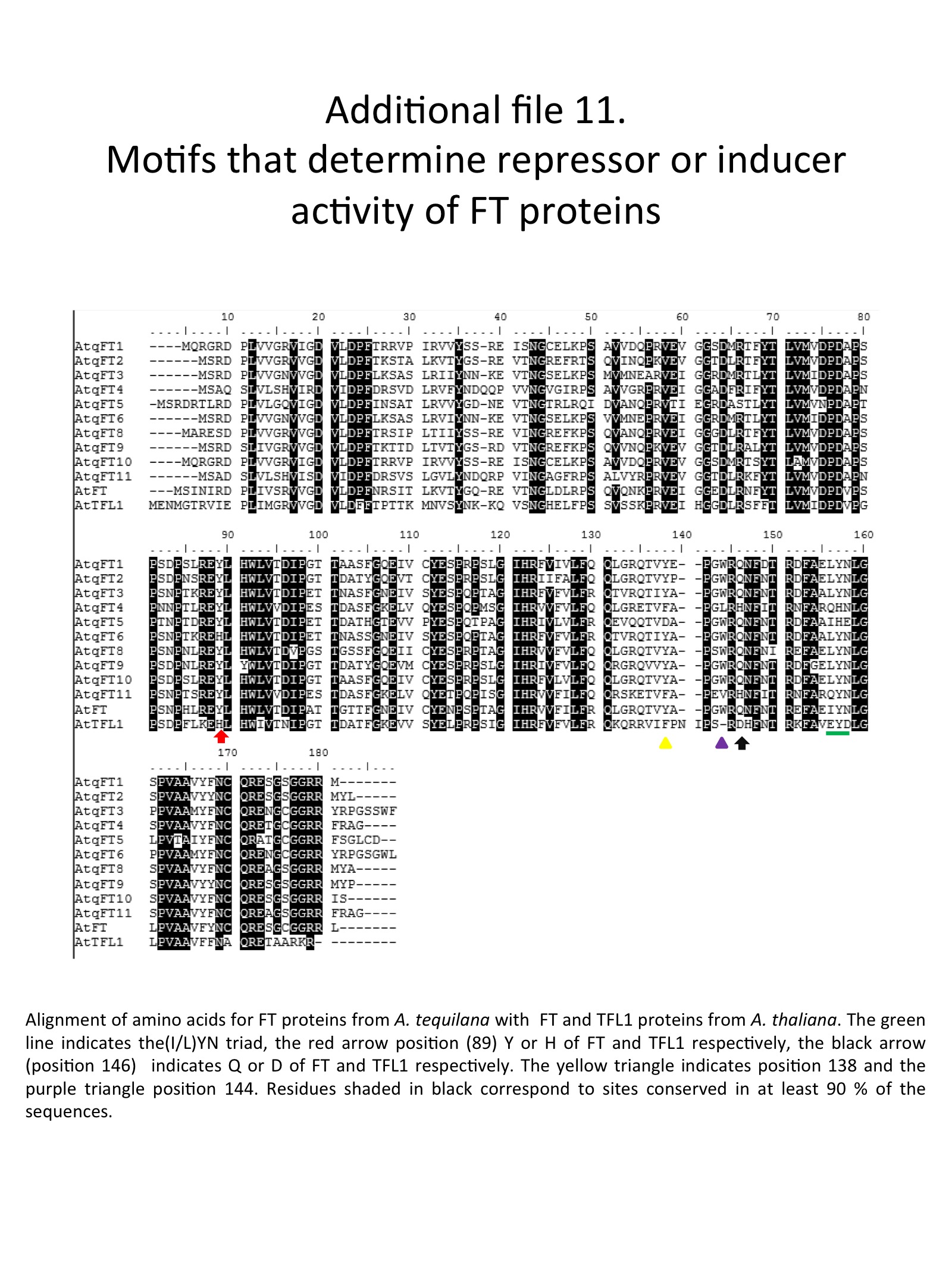

Supplement: Supplementary file 11 — Motifs that determine repressor or inducer activity of FT proteins. (JPG 711 kb) [file 12864_2019_5808_MOESM11_ESM.jpg]

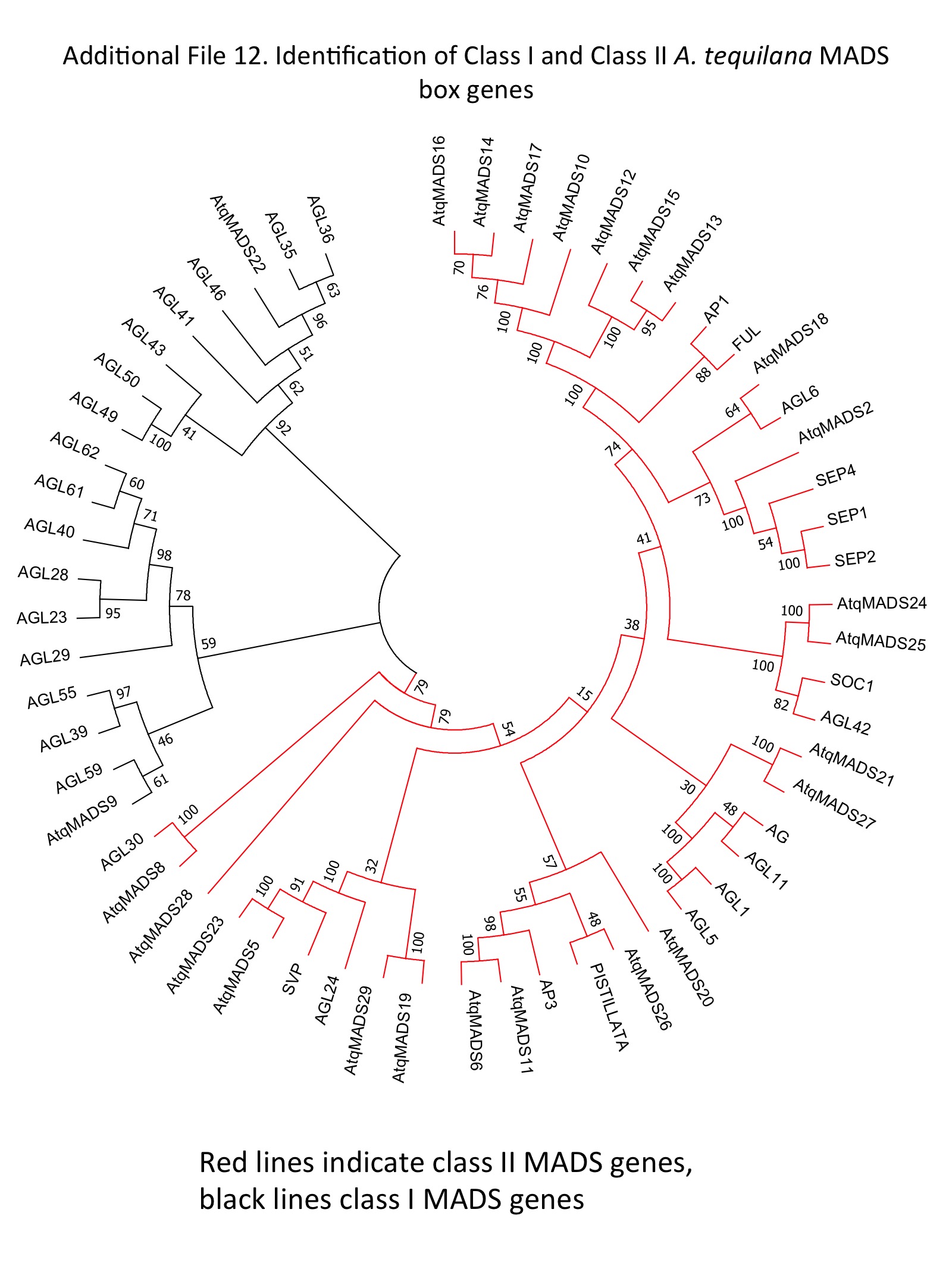

Supplement: Supplementary file 12 — Identification of Class I and Class II A. tequilana MADS box genes. (JPG 393 kb) [file 12864_2019_5808_MOESM12_ESM.jpg]
